# Single‐cell transcriptomics redefines focal neuroendocrine differentiation as a distinct prostate cancer pathology

**DOI:** 10.1002/1878-0261.70099

**Published:** 2025-07-24

**Authors:** Rosalia Quezada Urban, Shivakumar Keerthikumar, Ashlee Clark, Hong Wang, Belinda Phipson, Andrew Bakshi, Andrew Ryan, Heather Thorne, Renea A. Taylor, Mitchell G. Lawrence, Gail P. Risbridger, Roxanne Toivanen, David L. Goode

**Affiliations:** ^1^ Cancer Research Division Peter MacCallum Cancer Centre Melbourne Australia; ^2^ Sir Peter MacCallum Department of Oncology University of Melbourne Parkville Australia; ^3^ Department of Anatomy and Developmental Biology, Monash Biomedicine Discovery Institute Monash University Clayton Australia; ^4^ Bioinformatics Division The Walter and Eliza Hall Institute of Medical Research Parkville Australia; ^5^ Department of Medical Biology University of Melbourne Parkville Australia; ^6^ TissuPath Pathology Ltd Mount Waverley Australia; ^7^ Department of Physiology, Biomedicine Discovery Institute Cancer Program Monash University Clayton Australia; ^8^ Cabrini Institute Cabrini Health Malvern Australia; ^9^ Melbourne Urological Research Alliance Monash University Clayton Australia; ^10^ Department of Biochemistry and Molecular Biology Monash University Clayton Australia

**Keywords:** neuroendocrine prostate cancer, patient‐derived xenograft, single‐cell RNA sequencing, tumour heterogeneity

## Abstract

Neuroendocrine prostate cancer (NEPC) tumours are classified by pathology into several distinct subtypes. Gene expression profiling has revealed transcriptional heterogeneity across NEPC, but this is rarely considered in the context of variation between pathologies. Diagnosis typically relies on immunohistochemical markers (CHGA, SYP, NCAM1) and genomic alterations in RB1, PTEN and TP53. We hypothesized that NEPC pathologies have unique transcriptional features. Single‐cell RNA sequencing of 18 632 tumour cells from nine patient‐derived xenograft models representing five pathologies (small‐cell and large‐cell neuroendocrine carcinomas, focal neuroendocrine differentiation (Focal NED), low‐grade neuroendocrine and amphicrine) demonstrated pathway‐specific enrichment. Focal NED and amphicrine tumours exhibited cellular subpopulations enriched for KRAS, IL2‐STAT5 and TNF signalling pathways, absent in small‐ and large‐cell carcinomas, which were instead enriched for Myc and E2F pathways. Furthermore, focal NED cells exhibited minimal clonal divergence from adjacent adenocarcinoma cells, while small cell carcinoma cells were clonally distinct. These data underscore significant transcriptional variation among NEPC pathologies, highlighting focal NED's unique biological context and its clinical implications.

## Graphical abstract

Single‐cell transcriptomics of prostate cancer patient‐derived xenografts reveals distinct features of neuroendocrine (NE) subtypes. Tumours with focal NE differentiation (NED) share transcriptional programmes with adenocarcinoma, differing from large and small cell neuroendocrine prostate cancer (NEPC). Our work defines the molecular landscape of NEPC, revealing layers of transcriptional heterogeneity that provide a basis to develop new therapeutic opportunities for this subtype of prostate cancer.

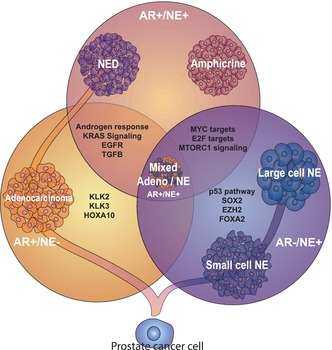

**Abbreviations**
AR: androgen receptor
Cx: castrated
Focal NED: focal neuroendocrine differentiation
NEPC: neuroendocrine prostate cancer
PDX: patient‐derived xenograft

## 1 Introduction

Neuroendocrine prostate cancer (NEPC) is diagnosed on the basis of immunohistochemistry (IHC) of canonical neuroendocrine cell surface markers such as chromogranin A (CHGA), synaptophysin (SYP) and CD56 (NCAM1) [1]. NEPC can arise via lineage plasticity under prolonged androgen deprivation, most often in metastatic lesions [2, 3], or occasionally *de novo* at diagnosis [4, 5]. No effective long‐term treatments exist for NEPC, and overall patient survival rates are very poor [3, 6].

Distinct neuroendocrine pathologies in prostate cancer have been defined by histology and morphology [2, 4, 6, 7]. The small‐cell and large‐cell neuroendocrine carcinomas most often associated with NEPC typically lack detectable androgen receptor (AR) signalling [4, 5]. In contrast, the amphicrine pathology displays strong co‐expression of both AR‐activated and neuroendocrine genes [4, 5]. Prostate adenocarcinoma with focal neuroendocrine differentiation (NED) displays small, scattered pockets of cells expressing neuroendocrine markers. Focal NED does not fully adhere to accepted definitions of NEPC [4, 5], and its influence on clinical outcomes remains uncertain [8]. The molecular foundations of diversity among neuroendocrine pathologies in prostate cancer continue to be elusive, contributing to suboptimal therapeutic outcomes [9].

Through discovery and profiling transcriptionally distinct cell populations, single‐cell RNA‐sequencing (scRNA‐seq) offers a way to directly characterize rare, dispersed pathologies such as focal NED and uncover molecular differences between subtypes of NEPC obscured by bulk sequencing. Prior scRNA‐seq studies revealed substantial intra‐tumoural heterogeneity at the transcriptional level within NEPC [10, 11, 12], but primarily focused on transdifferentiation from adenocarcinoma to NEPC. The links between transcriptional heterogeneity and the biological features and clinical presentation of varied NEPC pathologies remain much less well explored, however, in part due to difficulties sourcing tissues from rare pathologies.

To better understand underlying molecular differences between the diverse neuroendocrine pathologies that arise in prostate cancer, we turned to patient‐derived xenograft (PDX) models to capture a more extensive representation of pathologies than were included in previous studies. We performed scRNA‐seq on nine PDX models covering five distinct neuroendocrine pathologies of prostate cancer. Both the type and frequencies of transcriptionally distinct cellular subpopulations varied between PDXs of different pathologies. Focal NED cells displayed unexpected co‐expression of AR signalling and NE markers as well as differential patterns in oncogenic pathway expression, marking focal NED as a distinct molecular entity within the landscape of NEPC.

## 2 Methods

### 2.1 Patient‐derived xenografts

Patient‐derived xenografts (PDXs) were acquired from the Melbourne Urological Research Alliance (MURAL). PDXs are maintained in compliance with Monash University animal ethics approval (MARP 2014/085), as described previously [13]. Briefly, PDXs are passaged by sub‐renal or sub‐cutaneous grafting into 6–8‐week‐old immunocompromised male NOD.Cg‐Prkdc^*scid*^ Il2rg^*tm1Wjl*^/SzJ (NSG; RRID:IMSR_JAX:005557) mice, supplemented with 5 mm testosterone implants for mixed or amphicrine pathologies, or surgically castrated mice for pure NE/AR‐null pathologies. NSG mice were purpose bred at Monash Animal Research Laboratories (Monash University, Monash Breeding Colony approval number 15 160, 27 334 and 30 132). All animal handling and procedures were approved by the Monash University Standing Committee of Ethics in Animal Experimentation (approval number 17 944, 17 963, 28 911 and 41 088). All NSG mice were bred and housed under controlled temperature (22 °C) and lighting (12 : 12 h light–dark cycle) and were fed chow diet *ad libitum*.

### 2.2 Dissociation of patient‐derived xenografts

PDXs were harvested from host NSG mice and cut into 2 × 2 mm pieces using a scalpel. Tumour pieces were digested in 15 mL RPMI, penicillin/streptomycin containing 13 U LiberaseTM (Sigma‐Aldrich, St. Louis, MO, USA) and 3 mg DNase (Roche Diagnostics, Basel, Switzerland), for 1 h at 37 °C. Samples were disrupted with a pipette every 30 min during incubation to ensure suspension of cells. After cells were spun for 5 min at 1000 rpm, red blood cells were lysed using Red Cell Lysis buffer (Sigma‐Aldrich, St. Louis, MO, USA) for 1 min. Red cell lysis was stopped with RPMI with 10% FBS. Cells were then resuspended in PBS, 1 mm CaCl_2_, with 2% FBS and underwent negative selection for viable cells using the EasySep Dead Cell Removal kit (Miltenyi Biotec, Bergisch Gladbach, Germany), according to the manufacturer's protocol. After selection, cells passed through a 30 μm cell strainer (Miltenyi Biotec). Cell viability was assessed using Trypan Blue. Samples with cell viability > 80% were resuspended in PBS containing 2% BSA and proceeded to single‐cell analysis.

### 2.3 Single‐cell RNA‐sequencing library preparation

scRNA‐Seq was done on dissociated PDXs using the 10× Genomics Chromium Single Cell 3′ Library & Gel bead Kit V3.0, per the manufacturer's instructions (CG000183 Rev C). Approximately 5000 PDX cells were input per sample. After reverse transcription, barcoded cDNA was purified with SILANE Dynabeads and amplified through 11 cycles of PCR. SPRIselect purification was performed on an Agilent Bioanalyzer High Sensitivity chip to quantify the fragment size and concentration of the amplified cDNA. Libraries were sequenced on an Illumina NovaSeq6000 using 151 bp paired‐end reads.

### 2.4 Expression quantification for individual cells

Paired FASTQ files were aligned to the indexed GRCh38 human and mm10 mouse reference genomes using xenocell v1.0 [14]. Cells with < 10% mouse‐specific reads in XenoCell were considered human tumour cells. Transcripts were quantified using the Alevin (salmon Software v1.2.1) tool [15] by aligning against the GRCh38 transcriptome and imported into seurat v3.2.0 [16] in R V4.2.0 [17] for downstream analysis.

### 2.5 Identification and profiling of transcriptional subpopulations (within each PDX)

Standardized filtering criteria to exclude cells with low‐quality features was applied to all samples using seurat (v3.2.0), involving the exclusion of cells expressing fewer than 50 genes, those with fewer than 1000 genes, and sample‐specific variations, including a high mitochondrial transcript fraction (range 25–30%) and a high transcript count (range 40 000–100 000) (see Table S1). We used the DoubletFinder [18] algorithm to identify doublets in our single‐cell RNA‐sequencing dataset. Optimal parameters were determined, and doublets were classified and visualized, revealing a low doublet rate, with < 2.5% detected in all samples (Fig. S1).

### 2.6 Cell cycle phase identification

To ascertain cell cycle phase of individual cells, the ‘CellCycleScoring’ function was employed. Canonical cell cycle markers [19] were incorporated into Seurat, with a specific focus on features associated with the G2/M phase and markers indicative of the S phase. These elements were utilized as essential input parameters for the ‘CellCycleScoring’ function, which effectively scored and classified each cell into distinct phases, namely ‘S’, ‘G2/M’ and ‘G1’.

### 2.7 Normalization, scaling and feature identification

The SCTransform function was utilized for normalization, scaling and identification of high variable features [20]. Default parameters were applied. Subsequently, principal component analysis (PCA) was executed using the top 3000 most highly variable features. The determination of the appropriate dimension was facilitated by an Elbow plot in subsequent steps.

### 2.8 Clustering and visualization

To initiate the clustering process, the ‘FindNeighbours’ function in Seurat facilitated the construction of a nearest neighbour graph, utilizing default settings. Dimensions were then selected based on individual object (sample) characteristics. The ‘FindClusters’ function employed the shared nearest neighbour (SNN) approach to identify distinct clusters of cells, with default parameters utilized, and the resolution determined per sample. Visualization of clustered cells was achieved through the Uniform Manifold Approximation and Projection (UMAP) dimensional reduction technique using the ‘RunUMAP’ function, employing default settings and the previously selected dimensions.

### 2.9 Optimal cluster determination

The optimal number of clusters was determined using the clustree function from the R package clustertree [21]. Clustering trees were constructed from resolutions spanning 0 to 1 in increments of 0.1, and optimal resolutions were chosen for each sample. Visual inspection of trees was employed to choose the optimal resolution, based on differentially expressed markers per cluster and marker overlap across clusters at each resolution.

### 2.10 Differential gene expression analysis

Marker genes per cluster were identified using Seurat's ‘FindAllMarkers’ function with a negative binomial test. Parameters included a log fold change threshold of 0.25 and a minimum fraction of 0.25 for detected genes. Expression profiles were visualized logarithmically, and the top five differentially expressed genes were chosen based on the highest difference and average log fold change. Manual curation refined selections for unique markers.

For gene set enrichment analysis, parameters were adjusted to improve sensitivity and specificity: Log fold change threshold was set to 0, and the minimum fraction of detected genes was set to 0.

### 2.11 Gene set enrichment analysis

For analysis of individual PDXs, cancer signatures were downloaded from the CancerSEA database [22] and scored per cell using Seurat's ‘AddModuleScore’ function. The ‘Dotplot’ function was used to visualize the top enrichment patterns within each cluster.

For analysis of clusters in the integrated data set, the ‘msigdbr’ package was used to obtain the Hallmarks (H), Oncogenic (C6) and KEGG (CP) gene sets from Molecular Signatures Database (MSigDB) [23]. Enrichment analysis was conducted using the UCell method, which calculates a Mann–Whitney U statistic for each gene set within a ranked list of genes per cell. A cut‐off of 1500 ranked genes was applied to account for single‐cell RNA‐seq dropout effects. The final enrichment score is the complement of the U statistic, scaled by gene set size and the cut‐off. Visualization of enrichment values was performed using the ‘heatmapEnrichment’ function with the scale parameter, returning the scaled mean enrichment score for every cluster [24].

### 2.12 Benchmarking data integration methods

Three algorithms for integration of single‐cell RNA‐sequencing were evaluated: Seurat's canonical correlation analysis (CCA), LIGER's integrative non‐negative matrix factorisation (iNMF) and Cluster Similarity Spectrum (CSS) from Simspec (Fig. S2). CCA aims to identify matching cell pairs across datasets that are maximally correlated. These ‘anchors’ represent a similar biological state, weighted based on the overlap in their nearest neighbours. This creates a reference to transfer data and metadata from one experiment to another (see Methods below for parameters). iNMF provides a low‐dimensional space in which each cell is defined by one set of dataset‐specific factors; each factor usually corresponds to a biologically interpretable signal representing a particular cell type. iNMF aims to identify shared and dataset‐specific metagenes across datasets (see Methods below for parameters). CSS considers every cell cluster in each sample for integration as an intrinsic reference and represents each cell by its transcriptome similarities to clusters across samples (see Methods below for parameters).

To benchmark and compare, integration pipelines were benchmarked using data from two PDX models derived from MURAL patient 224 (Table S1). PDX 224R is a small cell NE mixed with adenocarcinoma grown in a testosterone supplemented with intact gonads, while PDX 224‐Cx originated from grafting PDX 224R into a castrated host to generate a castration‐resistant subline. We assessed the ability of each integration to correctly cluster together neuroendocrine cells from these two homologous PDX tumours from intact and castrate hosts while excluding the adenocarcinoma cells from PDX 224 into separate clusters. Four metrics of integration quality were used: silhouette coefficient, mixing metric and local structure metric. Details of quality metric calculation are provided later in this method section.

The CSS Simspec integration method was ultimately chosen due to its ability to segregate adenocarcinoma from neuroendocrine cells and overlapping common neuroendocrine populations while preserving transcriptional heterogeneity from each sample. Initial integration using Simspec showed the best separation between adenocarcinoma cells and neuroendocrine populations (Fig. S3, panel C1). No neuroendocrine cells were detected in the adenocarcinoma clusters. Metrics showed that the Simspec integration method was able to remove batch effect (AWS: 0.3, mix metric: 298) while preserving the local structure of the clusters (score: 0.34). Entropy scores were low in all clusters, meaning that no adenocarcinoma cells were mixed with NE cells in any cluster and vice versa (Fig. S4, panel C).

Further analysis was performed to ensure that the grouping was not based on any technical source of variation, such as transcript count and mitochondrial percentage. The mitochondrial percentage is < 20%, and clusters are not formed by a high or low mitochondrial gene content (Fig. S3, panel C2). Transcript count showed an even distribution of cells with ~3000 transcripts per cell (Fig. S3, panel C3); proliferative cluster showed a higher transcript count of ~5000 per cell, as expected (Fig. S5, panel B).

Furthermore, cancer signatures and differential gene expression analysis were evaluated on the integrated dataset to identify if heterogeneity was preserved (Fig. S5 panel D‐E). After integration, clusters with cancer signatures such as EMT, metastasis, angiogenesis, stemness and apoptosis were detected. Like cancer signatures, markers that previously characterized the heterogeneity of each tumour were preserved and detected across samples (Fig. S5, panel D). Clusters 0, 1 and 2 showed some pattern differences in the expression of specific markers, and this may be because of castration (Fig. S5, panel E).

### 2.13 Implementation of integration algorithms

#### 2.13.1 Seurat SCTransform

To integrate multiple datasets with Seurat, individual normalization of each sample was done before integration. The normalization function was ‘SCTransform’, based on regularized negative binomial regression. During normalization, 3000 variable features were selected for downstream analysis. After normalization, a list of the objects to integrate was created. Next, setting features to use when integrating multiple samples was done using ‘SelectIntegrationFeatures’. This function generates a vector of the selected top scoring ranked features that will be used in the next step. Then, the function ‘PrepSCTIntegration’ determines the features to use in the downstream integration procedure and ensures that sctransform residuals are present for all features that will be used as ‘anchors’ in all datasets. The next step was to find features that could be used as anchors; the function ‘FindIntegrationAnchors’ was used with standard parameters to create an anchor set object; this gene set will be used for integration. ‘IntegrateData’ was then used to integrate all datasets given the previously pre‐computed anchor set.

#### 2.13.2 Liger

The ‘Seurat Wrappers’ package was used to integrate Seurat objects with Liger [25]. Before integrating, the count matrices of each sample must be merged into one Seurat Object; cell IDs were added, and the ‘merge’ function from Seurat was used. The pre‐processing steps include normalization, finding variable features and scaling data. Normalization was done with the ‘NormalizeData’ function and finding variable features with ‘FindVariableFeatures’; both functions used default settings. To scale the data, the function ‘ScaleData’ from Seurat was used; data were split by sample and not centred. The integration process starts with running the integrative non‐negative matrix factorization on the normalized and scaled datasets using ‘RunOptimizeALS’. The k parameter was set to 20, and the object was split by sample (processed separately); the rest of the parameters were used as default. The next step is to use the ‘RunQuantileNorm’ function with the resulting factors to cluster cells jointly and perform quantile normalization by dataset, factor and cluster to integrate the datasets fully. Default parameters were used, and the object was split by sample. Louvain clustering was performed after integration using ‘FindNeighbours’ and ‘FindClusters’ from Seurat. The dimensional reduction was made using ‘RunUMAP’ from the Seurat package.

#### 2.13.3 Simspec (CSS)

A Seurat object is required to integrate single‐cell data using the cluster similarity spectrum (CSS) algorithm in the Simspec package [26]. The data were pre‐processed with Seurat, and normalization, finding variable features, scaling the data, PCA and dimensional reduction UMAP were needed before integration. The ‘cluster_sim_spectrum’ function was used to integrate the data; the correlation method used was ‘Pearson’, and the spectrum type used was ‘corr_kernel’, cluster resolution was set at 0.3, and the label tag was defined as sample names. After integration, dimensional reduction methods UMAP and PCA were run; the type of reduction used was ‘css’ and ‘css_pca’, respectively, and 10 dimensions were selected for each step. Then, the ‘FindNeighbors’ and ‘FindClusters’ functions were used to calculate clusters after integration. The resolution was set at 0.3, and 10 dimensions were used. Quality and suitability of data integration was evaluated by considering multiple factors and potential confounders, including cell cycle phase, transcript counts and mitochondrial and ribosomal percentages (Figs S3– S5).

#### 2.13.4 Metrics for evaluating integration performance

To compare the integration results, three measures of integration quality were used: the silhouette coefficient, a mixing metric and a local structure metric.

#### 2.13.5 Silhouette coefficient

The cluster R package (v2.1.4) was used to calculate the silhouette coefficient. Before computing the silhouette score, distances were calculated using the code from Stuart, 2019 [27]. The ‘Dist’ function from the package stats (v3.6.2) in R was used for the distance matrix computation. Distances were computed using the UMAP space defined by the two dimensions for all methods. Labels of clusters must be defined; the previous ID of each cluster in each sample was used as a label. Then, the silhouette score was calculated using the distance matrices previously calculated and the integer vector with the previous cluster labels. Then, the silhouette score was added to the metadata of the integrated Seurat object. A score of 1 or close to 1 represents dense and well‐separated clusters. Furthermore, a score of 0 or −1 corresponds to overlapping clusters. A higher score equals high performance.

#### 2.13.6 Mixing metric

Stuart et al. in 2019 designed the mixing metric [27]. It is constructed to evaluate how well mixed the input datasets were after integration. They reason that if neighbouring cells from a cell are well mixed, the closest neighbours should contain at least a small number (*k* = 5) of cells from each dataset. If poorly mixed, the cell will be surrounded by a small subset of datasets or only the cells from their dataset. The function of ‘MixingMetric’ in the Seurat R package was used. This function examines the local neighbourhood of each cell and determines for each group (clusters of datasets after integration) the k nearest neighbour and what rank that neighbour was in the overall neighbourhood. Then, it subtracts the median across all groups as the mixing metric per cell. For this analysis, the grouping variable was the previous ID, the reduction used was ‘umap’, the dimensions to use were 1 : 2, the k parameter was set to 5 and the max.k was set to 300; this was the suggested parameter by the user guide. For this metric, the higher the score, the better the integration.

#### 2.13.7 Local structure metric

It is a metric designed to measure how well each dataset's original structure (cluster) is preserved after integration. The function ‘LocalStruct’ splits the data back into its original dataset, re‐calculates the PCA on the uncorrected data and detects the *k* = 100 closest nearest neighbours. This function also computes the 100 nearest neighbours based on the PCA of the integrated dataset. Then, the intersection of these two neighbours for each cell is calculated, and the overlap fraction is computed. The higher the score, the better the performance.

#### 2.13.8 Entropy

To calculate the cluster sample diversity using Shannon Entropy, we used the Seurat R package (version 4.0.4) in R software. The function ‘calc_diversity’ was employed, which takes a Seurat object as input. The Seurat object should have columns indicating sample IDs and cluster IDs. The argument ‘sample_id’ specifies the name of the column containing the sample ID information, and ‘group_id’ specifies the name of the column containing the cluster ID information. We defined the ‘sample_id’ column as ‘orig.ident’, which indicates the original identity of the samples, and ‘group_id’ as ‘cell_type’, which represents the cells that are labelled as adenocarcinoma or neuroendocrine.

The Shannon Entropy method was applied to calculate the diversity of clusters across samples. Shannon Entropy is a commonly used metric for measuring diversity in a dataset. It provides a quantitative measure of the uncertainty or randomness of a system, in this case, the distribution of clusters across samples.

### 2.14 Downstream analysis for integrated dataset

Following integration, downstream analyses including differential gene expression (DGE) and gene set enrichment analysis were executed. DGE analysis was performed same as described above, utilizing the ‘FindAllMarkers’ function from the Seurat package. For gene set enrichment analysis (GSEA), MSigDB datasets were employed. Similar to the previous GSEA analysis on individual samples, the ‘fgseaMultilevel’ function was utilized for the enrichment analysis, employing default parameters.

### 2.15 Co‐expression analysis

Co‐expression analysis was conducted using the ‘Featureplot’ visualization function within Seurat, with the ‘blend’ argument set to TRUE. A co‐expression scale ranging from 0 to 10 was established, where 0 represents the lowest and 10 the highest expression. A maximum cut‐off value of q25 (quantile) was set to capture the minimum expression of markers. The ‘blend’ threshold was set to 0.1, initiating the blending of selected colours from the weakest signal. Normalized counts for each marker were used to calculate the co‐expression percentage across cells.

### 2.16 Scoring activity of transcription factor regulons with SCENIC

The integrated R object file containing raw counts matrix file was loaded into R, and gene regulatory networks were inferred using the pyscenic package (v0.11.2) [28]. Regulons with > 20 target genes were identified, and their activity was depicted in heatmaps.

### 2.17 Identification of clonal subpopulations

Clonal subpopulations were defined by chromosomal arm‐level copy‐number differences using Gaussian mixture models in a reference‐free manner [29]. Code was adapted from the module5_cna_subclones. R script available at https://github.com/gabrielakinker/CCLE_heterogeneity.

Libraries for whole‐genome sequencing (WGS) were prepared using the TruSeq DNA Nano High Throughput kit (Illumina) and sequenced as 150 bp paired‐end reads on a NovaSeq 6000 (Illumina). Reads from PDXs were aligned to hg19 (Ensembl Homo_sapiens.GRCh37.73.dna) and mm10 (Ensembl Mus_musculus.GRCm38.73.dna) using BWA MEM (v0.7.17), with duplicates marked by picard (v2.17.3). xenomapper (v1.0.1) [30] was used to identify reads mapping to hg19 only. The patient germline (blood) sample was aligned and processed in the same fashion but to the hg19 reference only.

PDX and germline BAM files sorted with samtools (v1.9) were an input for the HATCHet algorithm (v0.1) [31] for clonality assessment based on copy‐number alterations using Gurobi Optimizer v9.1.1 (Linux 64‐bit), parameters: mapQ = 11, baseQ = 11, snpQ = 11, minCov = 10, maxCov = 300, binSize = 100 kb and sensitivity (−l) of 0.4.

## 3 Results

### 3.1 Establishing patient‐derived models of neuroendocrine pathologies in prostate cancer

**Fig. 1:**
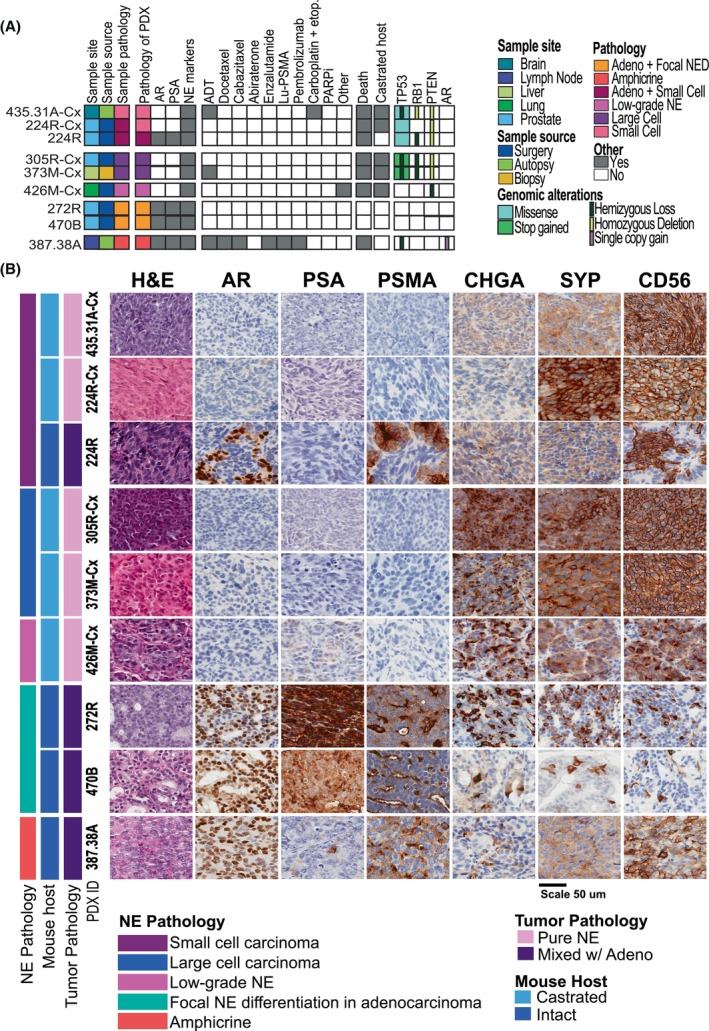
Diverse clinical and pathological landscape of MURAL PDXs with neuroendocrine features. (A) Clinical characteristics of the donor tumours used to establish of PDX models included in this study, the heatmap summarises the features of the patient samples, pathology of the PDXs, the patients' treatment histories, collection method, follow‐up, whether the PDXs are maintained intact NSG mice with testosterone implants or castrated (Cx) NSG mice and major genomic alterations. (B) Histopathology of PDX tumours, showing tissue morphology and staining for protein markers of adenocarcinoma (AR, PSA) and neuroendocrine (CHGA, SYP, CD56) Sidebar indicates assigned PDX tumour pathology and mouse host type. Scale bar = 50 μm. adeno, adenocarcinoma; ADT, androgen deprivation therapy; AR, androgen receptor; etop, etoposide; H&E, haematoxylin and eosin staining; Lu‐PSMA, lutetium prostate‐specific membrane antigen therapy; NE, neuroendocrine; NED, neuroendocrine differentiated; PARPi, poly ADP‐ribose polymerase inhibitors; PDX, patient‐derived xenograft; PSA, prostate‐specific antigen.

This study includes nine patient‐derived xenografts (PDXs) with neuroendocrine features from the Melbourne Urological Research Alliance (MURAL) collection: 8 published models [13] and one newly described PDX (470B). The selected PDXs represent adenocarcinoma with neuroendocrine differentiation (Focal NED; *n* = 2), amphicrine carcinoma (*n* = 1), mixed adenocarcinoma‐small cell (*n* = 1), small cell (*n* = 2), large‐cell prostate cancer (*n* = 2) and low‐grade neuroendocrine carcinoma (*n* = 1) (Fig. 1). Thorough histological assessment, genomic and transcriptomic profiling were conducted to determine pathology and confirm fidelity with neuroendocrine phenotypes of the original donor patients [13].

Four PDXs originate from radical prostatectomies of patients who had not received any systemic therapies (224R, 305R‐Cx, 272R, 470B). Another four PDXs originate from metastases via biopsy, metastasectomy or rapid autopsy from patients with prior treatment, including ADT, androgen receptor signalling inhibitors, taxane chemotherapy, platinum chemotherapy and Lu‐PSMA (435.31A‐Cx, 373M‐Cx, 426M‐Cx, 387.38A) (Fig. 1A). Notably, patient 426M‐Cx was diagnosed with *de novo* neuroendocrine prostate cancer before the age of 30, while patient 470B had a germline *BRCA2* mutation.

Tumour tissues were initially grafted into immunocompromised NSG mice with testosterone implants [13]. Several PDXs are maintained under these conditions (224R, 272R, 387.38A, 470B). Other PDXs were subsequently regrafted in castrated (Cx) NSG mice to simulate patients undergoing ADT (305R‐Cx, 373M‐Cx, 426M‐Cx, 435.31A‐Cx and 224R‐Cx) (Table S1). The tumour from patient 224 was split into two PDX sub‐lines. Growth in testosterone‐supplemented NSG mice yielded PDX 224 with mixed adenocarcinoma‐small cell pathology, while selection in castrated NSG mice resulted in PDX 224‐Cx with pure small cell pathology. Targeted exome sequencing revealed an abundance of alterations to *TP53*, *RB1* and *PTEN* in these PDXs, which is common in NEPC (Fig. 1A; Fig. S6).

### 3.2 Prostate cancer cells with neuroendocrine pathology include a diverse array of transcriptional states

Transcriptional profiles were obtained from individual cells from all nine PDXs using 10X Genomics Chromium Single Cell 3′ single‐cell RNA sequencing (Methods). After removing mouse cells and iterative filtering of low‐quality cells (Methods), 1202–7796 cells were detected per PDX (mean: 2659) (Table S1). The average number of genes detected per cell per PDX ranged from 2829–8037 (mean: 4869.6). This demonstrates the robust isolation of high‐quality tumour cells from prostate cancer PDXs.

Cells were clustered into subpopulations based on transcriptional differences and visualized on Uniform Manifold Approximation and Projection (UMAP) plots, with the optimal number of clusters per sample determined using Clustree (Methods). Each tumour contained 3–8 transcriptionally distinct subpopulations of cells, with an average of 5 subpopulations (Fig. S7). In the focal NED PDXs 272R and 470B, neuroendocrine clusters were located on the UMAPs in close proximity to adenocarcinoma clusters, but in the mixed adeno‐small cell PDX 224R, these two populations were clearly distant from one another (Fig. S7).

Functional enrichment analysis using the MSigDB Hallmarks and CancerSEA signatures [22, 23] revealed transcriptional subpopulations enriched for proliferation and stemness signatures in at least one cluster in every tumour, with EMT, hypoxia and invasion signatures also consistently appearing across all neuroendocrine pathologies in prostate cancer (Fig. S7). In contrast, the degree of transcriptional heterogeneity varied with tumour pathology. While most PDXs had 2–3 distinct neuroendocrine clusters, all small cell NE PDXs had 5–6 subpopulations regardless of origin in primary (224R and 224R‐Cx) or metastatic (435.31A‐Cx) tissues. Differences in clustering patterns across PDXs suggest each neuroendocrine pathology harbours its own set of transcriptionally distinct subpopulations.

### 3.3 Distinctive transcriptional subpopulations distinguish different neuroendocrine pathologies from each other

**Fig. 2:**
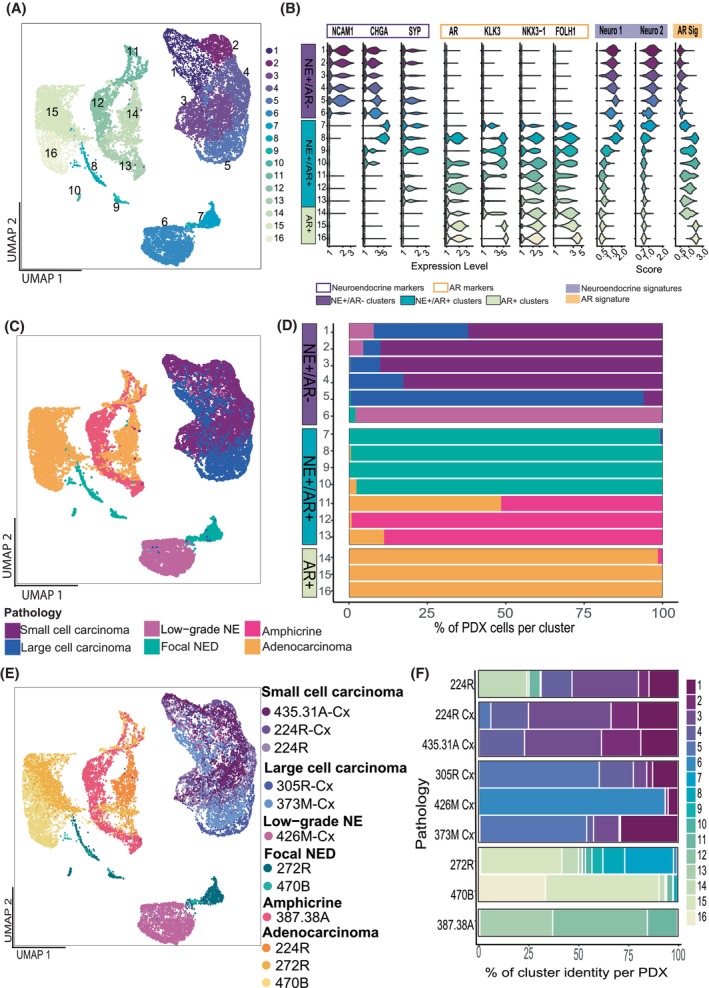
Inter‐tumoural heterogeneity can be observed between the different pathologies and patients. (A) UMAP depicting the multiple sub‐clusters detected in the integrated dataset. 16 clusters were detected. (B) Violin plot showing the range of expression of the neuroendocrine‐specific genes (SYP, CHGA, NCAM1) and androgen‐regulated genes (AR, KLK3, NKX3.1, FOLH1) per cluster. Clusters 1–6 are labelled as NE+/AR‐, clusters 7–13 are labelled as NE+/AR+ and clusters 14–16 are AR+/NE−. (C) UMAP shows the location of each sample. Clusters 1–6 comprise small and large cell pathologies. Clusters 7–13 include mixed pathologies (NED and amphicrine). Clusters 14–16 consists in adenocarcinoma cells. (D) Stacked bar plot representing the contribution of each tumour to the individual clusters. (E) UMAP coloured by PDX sample. (F) Stacked bar plot describing the proportion of clusters per PDX sample. AR, androgen receptor; PDX, patient‐derived xenograft; NE, neuroendocrine; NED, neuroendocrine differentiated. Data shown is representative of a single sample (*n* = 1).

To compare transcriptional subpopulations across NE pathologies, we integrated data from 18 632 cells from all 9 PDXs using CSS Simspec, which showed optimal ability to match cells based on pathology after benchmarking on the 224R and 224R‐Cx samples (Methods). The resulting 16 UMAP clusters revealed tumour pathology (Fig. 2A) had a greater influence on the clustering of cells on the UMAP than the cell cycle state, prior treatment status or site of tissue collection (Fig. S8).

Clusters 1–6 displayed robust expression of neuroendocrine markers NCAM1, CHGA and SYP and virtually no expression of AR signalling markers (Fig. 2B): thus, they were labelled NE+/AR‐ clusters. Cells in clusters 1–6 were predominantly from tumours with large or small cell NE pathologies (Fig. 2C,D). Clusters 1–4 were shared across PDXs with small cell pathology (224R, 224R‐Cx and 435.31A‐Cx) and large‐cell pathology (305R‐Cx and 373 M‐Cx) (Fig. 2E,F), revealing an overlap in the composition of some transcriptional subpopulations between these types of NEPC. In contrast, cluster 5 predominantly contained cells from large‐cell NEPC tumours (305R‐Cx and 373 M‐Cx). Clusters 1–5 contained a high proportion of cells in S and G2M phase, reflecting the highly proliferative nature of fully differentiated NEPC (Fig. S8A). Nearly all cells in cluster 6 were from PDX 426 M‐Cx, which has low‐grade NE pathology. This cluster was situated far from the other NE+/AR− populations, likely reflecting the unique clinical characteristics of the patient.

Clusters 7–13 co‐expressed neuroendocrine and AR signalling markers (NE+/AR+; Fig. 2B), with variable expression of CHGA and/or SYP and little to no NCAM1. Similarly, expression of AR and KLK3 varied across these clusters. This variability was reflected in the spatial heterogeneity of our focal NED tumours, as shown by staining of a representative whole tissue section from PDX272R and PDX470B (Fig. S9). FOLH1 (PSMA) expression was also observed in clusters 7–13, consistent with AR activity and absent from AR‐negative clusters. Most cells in NE+/AR+ clusters came from PDXs with intermediate focal NED or amphicrine pathologies (Fig. 2C,D), indicating focal NED is also an AR‐expressing neuroendocrine pathology. Clusters 7–10 were specific to tumours with focal NED pathology (PDX 272 and 470B) while clusters 11–13 were from the amphicrine tumour (Fig. 2E,F) indicating that despite shared AR expression and related AR activity genes such as FOLH1, focal NED and amphicrine pathologies diverge at the transcriptional level.

Finally, clusters 14–16 had robust expression of AR‐related genes with virtually no NE gene expression (AR+; Fig. 2B). They were comprised of cells from the adenocarcinoma component of the focal NED tumours and the mixed adeno‐small cell PDX 224R.

### 3.4 Cells with focal NED pathology co‐express neuroendocrine and adenocarcinoma markers

Detection of both AR signalling and neuroendocrine genes in clusters 8–10 could be due to cells having concurrent expression of both sets of genes, or indicate those clusters contained a mix of neuroendocrine and adenocarcinoma cells. To investigate, we performed cell‐level co‐expression analysis (Methods) to enumerate the fraction of cells with detectable expression of both markers AR signalling genes and canonical NE markers in the focal NED PDX 272R, along with the amphicrine PDX 387.38A and the mixed adeno‐small cell PDX 224R.

**Fig. 3:**
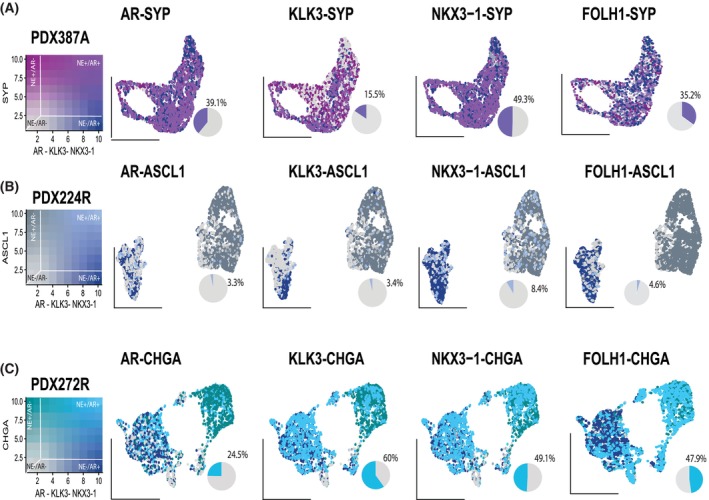
Co‐expression analysis of neuroendocrine and adenocarcinoma markers. Colour blending represents the co‐expression level; data have been scaled from 0–10. Zero represents cells without any expression of the markers, while 10 represents cells with the highest expression level. The percentage represents only the cells that co‐express such markers. (A) UMAPs representing the cells that co‐express AR‐SYP, KLK3‐SYP NKX3.1‐SYP and FOLH1‐syp in sample PDX387.38A. Pink represents cells expressing SYP, and dark blue represents cells expressing AR, KLK3, NKX3.1 or FOLH1. A purple shade represents cells that highly co‐express such markers. Grey shades represent cells that do not express any marker. (B) UMAPs representing the cells that co‐express AR‐CHGA, KLK3‐CHGA, NKX3.1‐CHGA and FOLH1‐CHGA in sample PDX272R. Green colour represents cells that uniquely express CHGA, and dark blue represents cells that uniquely express AR or KLK3 or NKX3.1. or FOLH1 A blue shade represents cells that highly co‐express such markers. Grey shades represent cells that do not express any marker. (C) UMAPs representing the cells that co‐express AR‐ASCL1, KLK3‐ASCL1, NKX3.1‐ASCL1 and FOLH1‐ASCL1 in sample PDX224R. Dark grey represents cells that uniquely express ASCL1, and dark blue represents cells that uniquely express AR or KLK3 or NKX3.1 or FOLH1. A light blue shade represents cells that highly co‐express such markers. Grey shades represent cells that don't express any marker. Data shown is representative of a single sample (*n* = 1).

As expected of an amphicrine tumour, PDX387.38A displayed strong co‐expression of *SYP* with multiple adenocarcinoma markers (Fig. 3A). Transcript counts for each pair of markers were generally robust in the cells where co‐expression was detected. In contrast, PDX 224R with the mixture of small cell and adenocarcinoma pathology displayed very limited co‐expression of its most abundant NE marker gene, *ASCL1* and AR signalling genes (Fig. 3B).

PDX 272R displayed robust expression of multiple neuroendocrine as well as adenocarcinoma markers (Fig. S10), particularly *CHGA*. *AR* and *CHGA* were concurrently expressed by 24.5% of cells in PDX 272R, while *KLK3‐CHGA* co‐expression was found in 60%, *NKX3.1‐CHGA* co‐expression in 49.1%, and FOLH1‐CHGA co‐expression in 47.9% (Fig. 3C), matching co‐expression rates of NE and AR markers seen in the amphicrine PDX 387.38A.

Interestingly, co‐expression of *CHGA‐* and *AR*‐regulated genes was also observed in the adenocarcinoma component of PDX 272R. However, cells from PDX 287R, a pure adenocarcinoma from the MURAL collection profiled in a recent study [32], displayed virtually no detectable expression of NE genes (Fig. S11). Co‐expression of both markers in prostate adenocarcinoma may therefore only occur in the context of focal neurodifferentiation.

### 3.5 Unique expression signatures distinguish types of neuroendocrine pathologies in prostate cancer

**Fig. 4:**
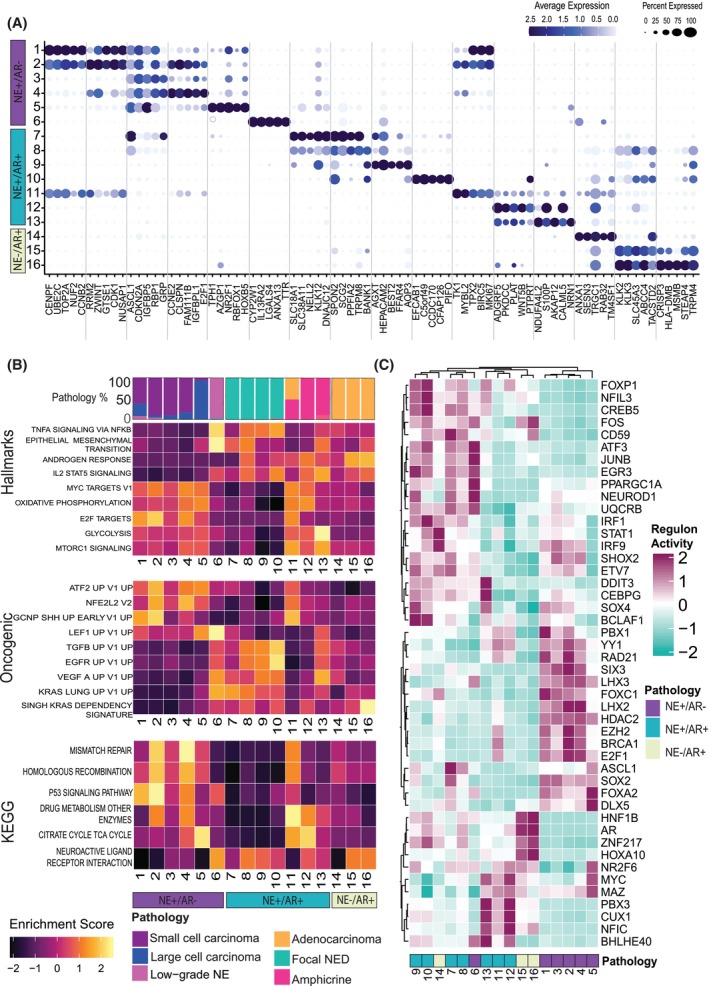
Characterising gene expression features that distinguish neuroendocrine subpopulations within the integrated data set. (A) Genes that are differentially expressed per cluster are shown. The size of the circle represents the percentage of cells expressing the gene. The intensity of the colour represents the gene's expression level; dark blue signifies a higher level, while light blue to white shows a low or null expression of the gene. (B) Enrichment of selected Hallmark, Oncogenic targets and KEGG gene sets from MSigDB are shown. The enrichment score is represented by colour; yellow is a positive enrichment score, and dark purple is a negative enrichment score. (C) Transcription factor analysis heatmap. Regulon activity is scored from 2 to −2, where 2 represents a positive regulon activity, and −2 symbolises a negative regulon activity. Here 46 manually curated TFs out of the 87 significantly enriched TFs reported by SCENIC are displayed. AR, androgen receptor; NE, neuroendocrine; NED, neuroendocrine differentiated; PDX, patient‐derived xenograft. Data shown is representative of a single sample (*n* = 1).

To identify transcriptional differences between neuroendocrine pathologies, we applied differential gene expression and gene set enrichment (GSE) analysis to all 16 clusters in the integrated data set. Very few differentially expressed genes (DEGs) were shared between the NE+/AR− clusters and the NE+/AR+ clusters (Fig. 4A), suggesting AR‐expressing neuroendocrine pathologies have distinct transcriptional signatures from those that lack AR expression. The exceptions were cell cycle genes such as *MKI67*, which overlapped between clusters 1, 2 and 11 due to their proliferative nature.

To find differences in cancer‐related pathways and processes between neuroendocrine pathologies, cluster‐level enrichment was assessed for the Hallmarks 50, oncogenic and Kyoto Encyclopedia of Genes and Genomes (KEGG) gene sets from MSigDB [23]. Cells from AR+/NE− small‐ and large‐cell neuroendocrine pathologies have distinct enrichment profiles from AR+/NE+ amphicrine and focal NED cells (Fig. 4B). Many gene sets showed mutually exclusive patterns of enrichments in focal NED as compared to small and large‐cell pathologies, while clusters from the amphicrine PDX shared gene set signatures with both focal NED and small and large‐cell PDXs.

Common enriched gene sets in the NE+/AR‐ clusters 1–5 included E2F targets and oxidative phosphorylation, consistent with the highly proliferative nature of small‐ and large‐cell NEPC. Noteworthy oncogenic signalling enrichments included MYC, MTORC1, LEF1, a key regulator of epithelial–mesenchymal transition (EMT) [33].

The NE+/AR+ clusters 7–10 dominated by focal NED cells displayed markedly different enrichments from clusters 1–5, including TNFA signalling via NFKB and KRAS signalling, which were also enriched in the adenocarcinoma clusters 14–16 but down‐regulated in the small/large cell clusters (Fig. 4B). In contrast to adenocarcinoma, there was high expression of genes up‐regulated by EGFR and TGFβ. Like in clusters 1–5, enrichment of EMT was seen, but in concert with enrichment of KRAS signalling rather than LEF1, indicating that in focal NED, activation of EMT may occur through KRAS instead [34]. Indeed, each pathology shows divergent expression of EMT genes (Fig. S12). Androgen response genes were most strongly up‐regulated in cluster 8, indicating AR signalling expression varies amongst focal NED cells.

Interestingly, clusters 11–13 primarily represent cells from the amphicrine PDX 387.38A, shared enrichment signatures with clusters from the NE+/AR‐ small‐ and large‐cell NE pathologies, and the NE+/AR+ focal NED cells (Fig. 4B). Cluster 11 shared a GSE profile with clusters 1–5, cluster 12 matched all the other neuroendocrine pathologies, while cluster 13 was most similar to focal NED as well as adenocarcinoma cells (clusters 14–16). Thus, the amphicrine PDX 387.38A contains a mix of transcriptional states covering both AR‐expressing pathologies as well as AR‐suppressed neuroendocrine pathologies.

Cluster 6, comprised nearly entirely of cells from the low‐grade NE PDX 426M‐Cx, had a GSE profile more similar to clusters 7–10 than to clusters 1–5, including enrichments for TNFA signalling via NFKB, EMT, KRAS signalling, and KEGG neuroactive ligand‐receptor interactions. Despite virtually no AR expression, PDX 426 M‐Cx appears to share characteristics with AR‐expressing focal NED.

Transdifferentiation to neuroendocrine prostate cancer is driven by master regulator transcription factors (TFs) [35, 36]. To infer how changes to gene regulatory networks contribute to differences in gene expression states across the 16 cell clusters in our data, we scored the activity of transcription factor regulons in each cluster using SCENIC [28]. Concordant with prior results, the small/large cell clusters (1–5) and focal NED clusters (7–10) showed clear divergence in inferred TF regulon activity (Fig. 4C). Clusters 1–5 were predicted to have high activity of several TFs that regulate proliferation, chromatin state and DNA replication/repair, including E2F1, EZH2, HDAC2 and BRCA1. Regulons for known neuroendocrine lineage regulators ASCL1, SOX2 and FOXA2 were active in all small/large‐cell and focal NED clusters but scored markedly higher in the latter. Numerous TFs had high activity in focal NED clusters 7–10 but weak to no activity in the small/large cell clusters (Fig. 4C), including stemness factors FOS and JUNB, and NEUROD1, a TF with variable expression across NEPC [37]. Focal NED clusters also showed overlap in TF activity with adenocarcinoma populations (clusters 14–16), aligning with retained AR signalling in those cells. As expected, AR was a shared TF, but there were also others, including FOS and the lineage factor HOXA10. HOXB13 was detected in clusters 6–16 (Fig. S13), but only clusters 7–16 co‐expressed AR and FOLH1 (PSMA), suggesting that HOXB13 alone does not control PSMA expression in our models. Cluster 6 (low‐grade NE) expressed HOXB13 but lacked both AR and PSMA (Fig. S13; Fig. 2A). As in the GSE analysis, TF activity in the amphicrine clusters 11–13 overlapped with the small/large cell and focal NED clusters, although ASCL1 was not active in amphicrine clusters.

**Fig. 5:**
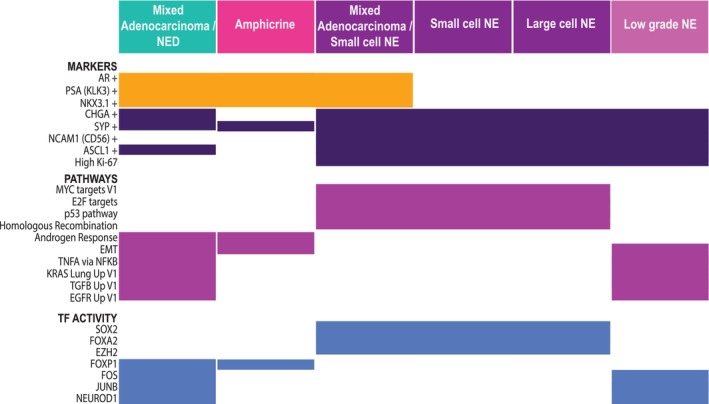
Summary of key transcriptional findings across NEPC pathologies. Summary chart depicting the expression of markers, pathways and transcriptional factors detected across NEPC pathologies. AR, androgen receptor; EMT, epithelial–mesenchymal transition; NE, neuroendocrine; NED, neuroendocrine differentiated.

In summary, several biologically meaningful expression differences beyond AR signalling were seen between the AR‐expressing focal NED and amphicrine pathologies and small and large cell neuroendocrine pathologies, which lack AR activity (Fig. 5). These included numerous pathways and processes involved in oncogenic signalling, inflammation and metastasis.

### 3.6 Single‐cell copy‐number profiling indicates focal NED and small cell neuroendocrine carcinoma arise by different means in tumours of mixed pathology

Our single‐cell transcriptional profiling supports the small cell neuroendocrine pathology as being further diverged from adenocarcinoma than the focal NED pathology. This view is further supported by pseudotime analysis (Fig. S14).

To determine whether clonal divergence exists between the adenocarcinoma and neuroendocrine subpopulations in PDXs 224R and 272R, we inferred copy‐number status at the chromosome arm level from the transcriptomes of individual cells. Following the methods of Kinker et al. (2020) [29], combined expression of genes on the same chromosome arm was measured in each cell to detect heterogeneity in expression level, chromosome arms indicative of sub‐clonal copy‐number gains and losses (Methods).

**Fig. 6:**
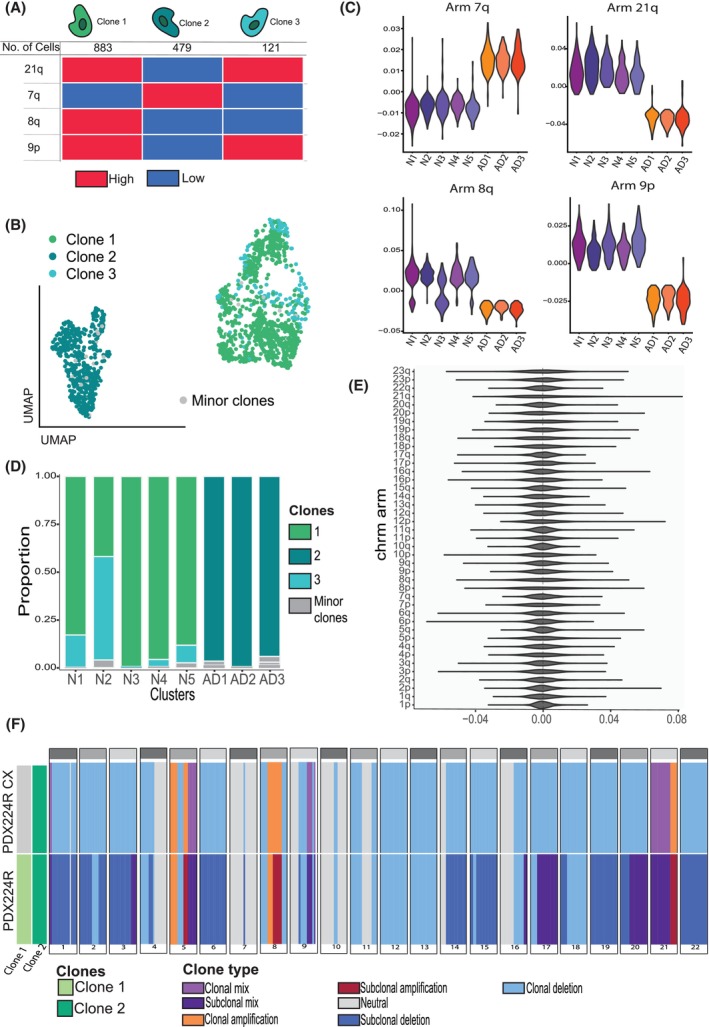
The small cell neuroendocrine and adenocarcinoma components of the mixed pathology in PDX224R comprise genetically distinct clonal subpopulations. (A) Inferred copy‐number profiles based on single‐cell RNA‐seq data across four chromosome arms (7q, 8q, 9p and 21q) identify three genetically distinct clones in PDX224R (Clone 1, 2 and 3). Heatmap indicates relative expression per arm (red = high, blue = low). (B) UMAP showing cells from PDX224R coloured by clone identity. Clone 2 localizes predominantly within adenocarcinoma clusters, while Clones 1 and 3 localize within neuroendocrine clusters. (C) Violin plots showing chromosome arm‐level copy‐number signal for each cell, stratified by clone, across arms 7q, 8q, 9p and 21q. (D) Proportional representation of each clone across transcriptionally defined clusters. Neuroendocrine clusters (N1–N5) are dominated by Clones 1 and 3, whereas adenocarcinoma clusters (AD1–AD3) are composed mainly of Clone 2. (E) Violin plots showing the distribution of copy‐number signal across all chromosome arms in PDX272R, indicating no evidence of distinct genetic subclones. (F) Stacked bar plot showing clonal composition and copy‐number alteration patterns across chromosomal arms in PDX224R and PDX224R‐Cx, as inferred from whole‐genome sequencing. Each vertical bar represents a chromosome arm, with segments denoting clone identity (Clone 1, Clone 2, or Minor clones) and clone type (e.g. clonal or sub‐clonal amplification/deletion, clonal mix). The data reveal loss of clonal diversity after castration, with PDX224R‐Cx retaining the neuroendocrine‐associated clone identified in the single‐cell RNA‐seq analysis of PDX224R. 7q, long arm of chromosome 7; 8q, long arm of chromosome 8; 9p, short arm of chromosome 9; 21q, long arm of chromosome 21; AD, Adenocarcinoma cluster; Cx, Castrate; N, Neuroendocrine cluster; PDX, Patient‐derived xenograft. Data shown is representative of a single sample (*n* = 1).

Three distinct clonal subpopulations were detected in PDX 224R on the basis of inferred copy‐number states on four chromosome arms: 21q, 8q and 9p and 7q (Fig. 6A). Clones 1 and 3 mapped exclusively to the neuroendocrine cell clusters of 224R, while Clone 2 was found only in the adenocarcinoma clusters (Fig. 6B,D). Expression of genes on 21q, 8q, 7q and 9p showed consistent differences across all neuroendocrine and adenocarcinoma clusters (Fig. 6C), indicating the neuroendocrine and adenocarcinoma cells in PDX 224R come from genetically distinct clones.

In contrast, cells from PDX 224‐Cx, which was derived from 224R via growth in castrate host mice, showed no substantial differences in gene expression at the chromosome arm level (Fig. 6E), suggestive of selection of a dominant clone from the neuroendocrine population post‐castration. To validate these findings, whole‐genome sequencing (WGS) to 80–90× coverage (Methods) of 224R, 224R‐Cx, and a germline sample from patient 224 was performed and analysed using HATCHet to infer clonal subpopulations based on copy‐number alterations [31]. HATCHet predicted two distinct clonal subpopulations in 224R differing in copy‐number profile across 17 of the 22 autosomes, including copy‐number changes on 7q, 8q, 9p and 21p (Fig. 6F). In contrast, only a single clone was detected in PDX 224R‐Cx. Patterns of amplification and deletion on 7q, 8q, 9p and 21p were concordant with those inferred from single‐cell RNA‐sequencing data.

In contrast, no differences in expression at the chromosome arm level were detected from the 272R single‐cell RNA‐sequencing data, suggesting this PDX most likely harbours only a single dominant clone (Fig. S15) comprising both the adenocarcinoma and focal NED populations. Presence of a cluster with low expression of both AR and NE markers between the adenocarcinoma and focal NED clusters marks 272R as potentially actively undergoing transdifferentiation (Figs S9, S10). PDX 470B also has focal NED pathology and likewise showed no evidence for copy‐number differences amongst adenocarcinoma and focal NED cells (Fig. S15). On the other hand, the genetic differences between the adenocarcinoma and small cell neuroendocrine subpopulations in PDX 224R are more consistent with divergence of two populations in the prostate prior to diagnosis. These contrasting patterns of sub‐clonality align with the concept that focal NED and small cell neuroendocrine pathologies are distinct entities, with focal NED being less diverged from adenocarcinoma.

## 4 Discussion

Integrative transcriptional analysis of 18 632 cells from nine PDXs with neuroendocrine features demonstrated that pathology is closely associated with gene expression in NEPC. Strikingly, focal NED cells retain expression of AR signalling genes at levels comparable to amphicrine and adenocarcinoma cells, while maintaining robust expression of neuroendocrine markers (Fig. 2). Widespread co‐expression of CHGA along with one or more of AR, KLK3, FOLH1 and NKX3.1 in PDX272 (Fig. 3) indicates that focal NED cells have the capacity for AR signalling. In line with this, PSMA expression in our cohort was restricted to NE+/AR+ cells, specifically in focal NED and amphicrine tumours, with co‐expression of PSMA and neuroendocrine markers only observed in these transitional phenotypes. This aligns with studies linking PSMA expression to AR activity and not with AR‐negative NEPC [38].

Degree of AR signalling across pathologies corresponded to differences in expression of multiple other oncogenic processes as well. The AR‐null small and large cell NE pathologies displayed strong expression of growth‐associated processes such as Myc signalling, DNA repair and oxidative phosphorylation (Fig. 4B). In contrast, focal NED, amphicrine and the low‐grade NE subpopulations were instead enriched for other pathways, including KRAS, TNF‐alpha and EGFR signalling. Similarly, inferred activity of master regulator transcription factors differed between pathologies (Fig. 4C).

Certain states along the trajectory from adenocarcinoma to NEPC captured in prior studies were evident in our data. Notably, focal NED cells showed the highest levels of IL2‐STAT5 signalling and activity of *STAT1* and *IRF1* (Fig. 4), inflammatory signalling pathways posited to drive lineage plasticity in prostate cancer [39]. Focal NED cells also displayed concurrent activity of *ASCL1* and *NEUROD1* (Fig. 4C), two transcription factors more often seen in separate subpopulations in NEPC [40, 41]. Along with dual expression of NE and AR genes, this may indicate focal NED as a more plastic and/or transitional state.

However, whether focal NED transitions to small or large cell pathology remains unresolved. The neuroendocrine and adenocarcinoma subpopulations within focal NED PDXs 272R and 470B appeared to originate from a common clone. In contrast, we clearly distinguished genetically distinct clones for the neuroendocrine and adenocarcinoma subpopulations of the mixed small cell‐adenocarcinoma PDX 224R (Fig. 6), indicating long‐standing divergence. Cheng et al identified rare neuroendocrine cells in adenocarcinomas from CRPC patients, but these expressed E2F1, SOX2 and YY1, markers we found to be up‐regulated in small and large cell pathology. Further longitudinal studies of PDXs and patients may shed light on whether focal NED is a transitional or terminally differentiated state.

Current standard of care chemotherapies do not confer lasting benefit in NEPC. Our results suggest focal NED may retain sensitivity to androgen‐targeting agents and could respond to disruption of KRAS and EGFR signalling. In contrast, Wnt targeting and other agents may work better against small‐ and large‐cell NEPC tumours.

## 5 Conclusion

Single‐cell RNA‐sequencing of a diverse spectrum of PDX models of NEPC reveals focal NED as being transcriptionally distinct from small‐ and large‐cell NEPC, requiring its own treatment and management strategies. Our work redefines the molecular landscape in NEPC, revealing previously hidden layers of transcriptional heterogeneity that provide a basis to further develop new therapeutic opportunities for this low‐survival subtype of prostate cancer.

## Conflict of interest

None of the authors have any conflicts of interest to declare.

## Author contributions

Conceptualization and study design: RQU, RT, DLG. Data acquisition (PDX generation, sample preparation): AC, HW, HT. Data analysis and interpretation: RQU, SK, AB, DLG, RT, MGL, GR, BP. Pathological review and classification: AR. Figure preparation: RQU, SK, AC, RT. Methodology review: BP. Manuscript writing: RQU, DLG. Manuscript revision and editing: RQU, RT, DLG, MGL, GR, BP, HT. Supervision: RT, DLG, MGL, GR. Final approval of manuscript: All authors.

## Peer review

The peer review history for this article is available at https://www.webofscience.com/api/gateway/wos/peer‐review/10.1002/1878‐0261.70099.

## Supporting information


**Fig. S1.** Doublet detection. (A) Percentage of doublets detected per sample. (B) Visualization of doublets in a UMAP per sample.

**Fig. S2.** Workflow chart of benchmarking process. (A) Seurat integration workflow process. Seurat has two ways of pre‐processing the data for integration; standard workflow and SCTransfrom. Standard workflow includes normalization, finding variable features and scaling data. SCTransfrom is a wrapper that includes the standard workflow, but the data is not scaled. (B) Liger integration workflow process. Integrative non‐negative matrix factorization (iNMF) method was used. (C) Simspec integration workflow process. The cluster similarity spectrum method was used. Two correlation methods might be used: Pearson or spearman. Pearson correlation uses kernel normalisation and Spearman correlation uses Z‐transformation normalisation.

**Fig. S3.** Integration outcomes of 3 different pipelines. UMAPS represents the outcome of each step of the process (A) Seurat pipeline. (A1) UMAP representing the Seurat integration using SCTransform as a normalisation method. In a green outline, castrated cells cluster with adenocarcinoma. (A2) UMAP shows the main subpopulation of castrated cells that cluster with adenocarcinoma. (A3) UMAP represents the integration after removing the cell cycle phase as a source of variation. Castrated cells from cluster N3 remain clustering with adenocarcinoma and are outlined in green. (A4) UMAP represents the outcome of integration with Seurat. (B) Liger pipeline. (B1) UMAP representing the Liger integration. In a green outline, castrated cells cluster with adenocarcinoma. (B2) UMAP shows the main subpopulation of castrated cells that cluster with adenocarcinoma—outlined in green castrated N2 cells. (B3) UMAP represents the integration after removing the cell cycle as a source of variation. Castrated N1 and N3 cells clustered with adenocarcinoma, outlined in green. (B4) UMAP showing the outcome of integration with Liger. C) Simspec CSS pipeline. (C1) UMAP represents the initial outcome of the integration using Simspec. Circled in a green outline, a cluster is uniquely formed by castrated cells. (C2) UMAP coloured by the expression of mitochondrial percentage. (C3) UMAP coloured by the transcript (UMI) count. (C4) UMAP with the Simspec integration pipeline after QC.

**Fig. S4.** Metrics for evaluating integration performance. (A) Local structure and Mix metric depicted in a dotplot. (B) Boxplot of AWS scores per cell resulting from each integration method. (C) UMAPs depicting cell type (Adenocarcinoma and neuroendocrine), clusters, and entropy score. Violin plot showing the score per cluster.

**Fig. S5.** Tumour heterogeneity is preserved after integration with CSS. (A) UMAP representing the integration of PDX224R and PDX 224R‐Cx. (B) UMAP depicting proliferative cells. A score close to one indicates a high expression of proliferative genes. (C) UMAP showing the 10 clusters formed after integration. Clusters were defined with the same method used on previous chapter. (D) Cancer CEA, NEURO I‐II, Squamous and AR responsive signatures. The ratio of the circle represents the proportion of cells that express such signature, the colour of the circle represents the level of expression, yellow represents a high expression, purple represents a low expression. (E) Heatmap showing the top markers per cluster.

**Fig. S6.** Mutational profiles of the patient‐derived xenograft models used in this study. (A) Protein‐coding mutations in selected cancer driver genes for each PDX model, coloured by effect on protein sequence. Sidebar displays overall frequency of mutations to each gene across the 9 PDXs. (B) Copy‐number aberrations in selected cancer driver genes for each PDX model coloured by magnitude of change relative to diploid.

**Fig. S7.** Functional state enrichment and transcriptional classification of single‐cell clusters across NEPC PDX models. UMAPs displaying cell clusters identified in each PDX, with corresponding gene set enrichment scores for functional states from the Cancer Single‐cell Expression Atlas (CSEA) (Yuan et al., 2018) and androgen receptor (AR) signaling and neuroendocrine prostate cancer (Neuro1 and Neuro2) signatures from Labrecque et al. (2019). Clusters are labelled according to whether cells express neuroendocrine markers (N), adenocarcinoma markers (AD) or both sets of markers (AM). PDXs are divided according to pathology. (A) mixed small cell‐adenocarcinoma PDX; (B) Small cell neuroendocrine prostate cancer (NEPC) PDX 224R‐Cx; (C) Small cell NEPC PDX 435.31A‐Cx; (D) Large cell NEPC PDX 305‐Cx; (E) Low‐grade neuroendocrine PDX 426M‐Cx; (F) Large cell NEPC PDX 373M‐Cx; (G) Adenocarcinoma with neuroendocrine differentiation (Adeno‐NED) PDX 272R; (H) Adeno‐NED PDX 470B; (I) Amphicrine PDX 387.38A.

**Fig. S8.** UMAPs of integrated data from all 9 PDXs with cells coloured by conditions. (A) Cell cycle phase, (B) site of collection or (C) treatment status.

**Fig. S9.** Whole tissue section to illustrate the ratio of NED vs adenocarcinoma. Scale bar = 500 μm.

**Fig. S10.** Expression of neuroendocrine markers genes in PDX 272R. Neuroendocrine and the adenocarcinoma marker genes expression by cluster in PDX 272R.

**Fig. S11.** Co‐expression of the androgen receptor (AR) and synaptophysin (SYP) genes in tumour cells from PDX 287R.

**Fig. S12.** Expression of genes from the epithelial–mesenchymal transition gene set. Gene set from the cancer single‐cell expression atlas (Cancer SEA) across UMAP clusters from the integrated analysis of PDX data.

**Fig. S13.** Expression of HOXB13 across clusters.

**Fig. S14.** Pseudotime trajectory (Monocle 2) for tumour cells in PDX 272. Trajectory begins in the adenocarcinoma component (left; yellow/orange) and continues into the neuroendocrine component (right; blue/purple).

**Fig. S15.** Relative expression of genes on each chromosome arm for tumour cells from PDX 470B.

**Table S1.** Summary of single‐cell RNA‐sequencing data for each PDX tumour included in this study.

## Data Availability

R and Python code used to analyse data and generate figures included in this manuscript is available at https://github.com/dlgoode/QuezadaUrban_scRNAseq. Raw single‐cell RNA‐sequencing reads have been deposited in dbGAP repository phs003369.v2.p1. Transcript counts and processed data are available from the corresponding author on reasonable request.
