# Supplementary figures and images for: Single‐cell transcriptomics redefines focal neuroendocrine differentiation as a distinct prostate cancer pathology
Source: Mol Oncol. 2025 Jul 24;19(10):2776–96. doi: 10.1002/1878-0261.70099 (PMC12515717; doi:10.1002/1878-0261.70099)

Percentage of Singlet and Doublet Cells per Sample

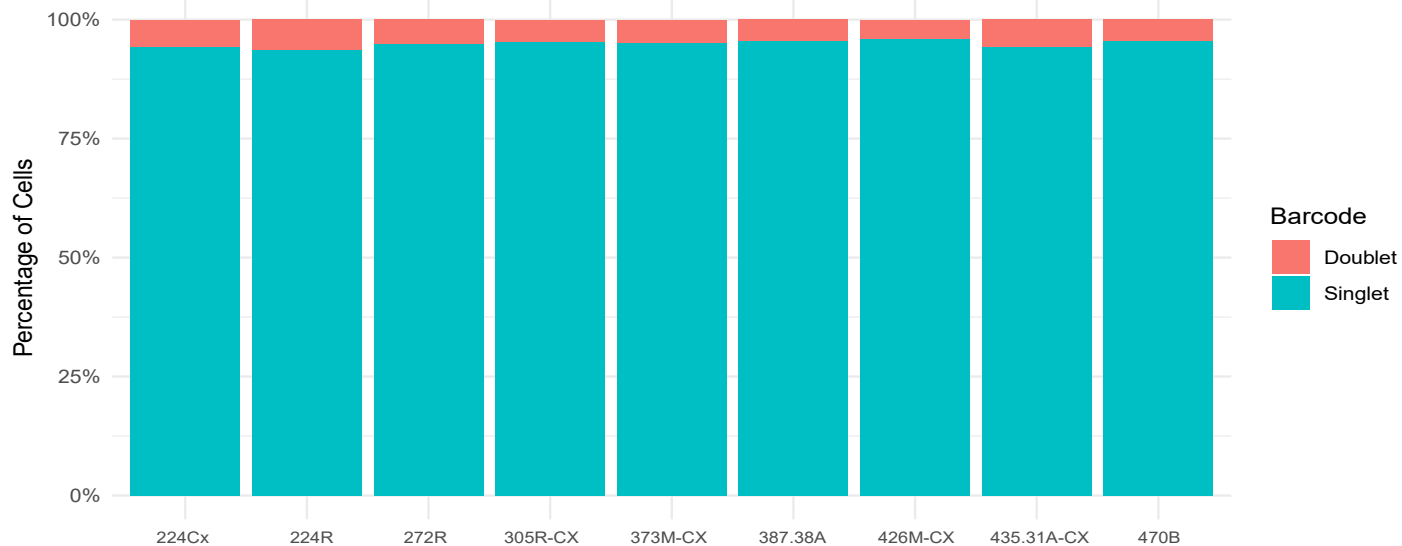

**B**

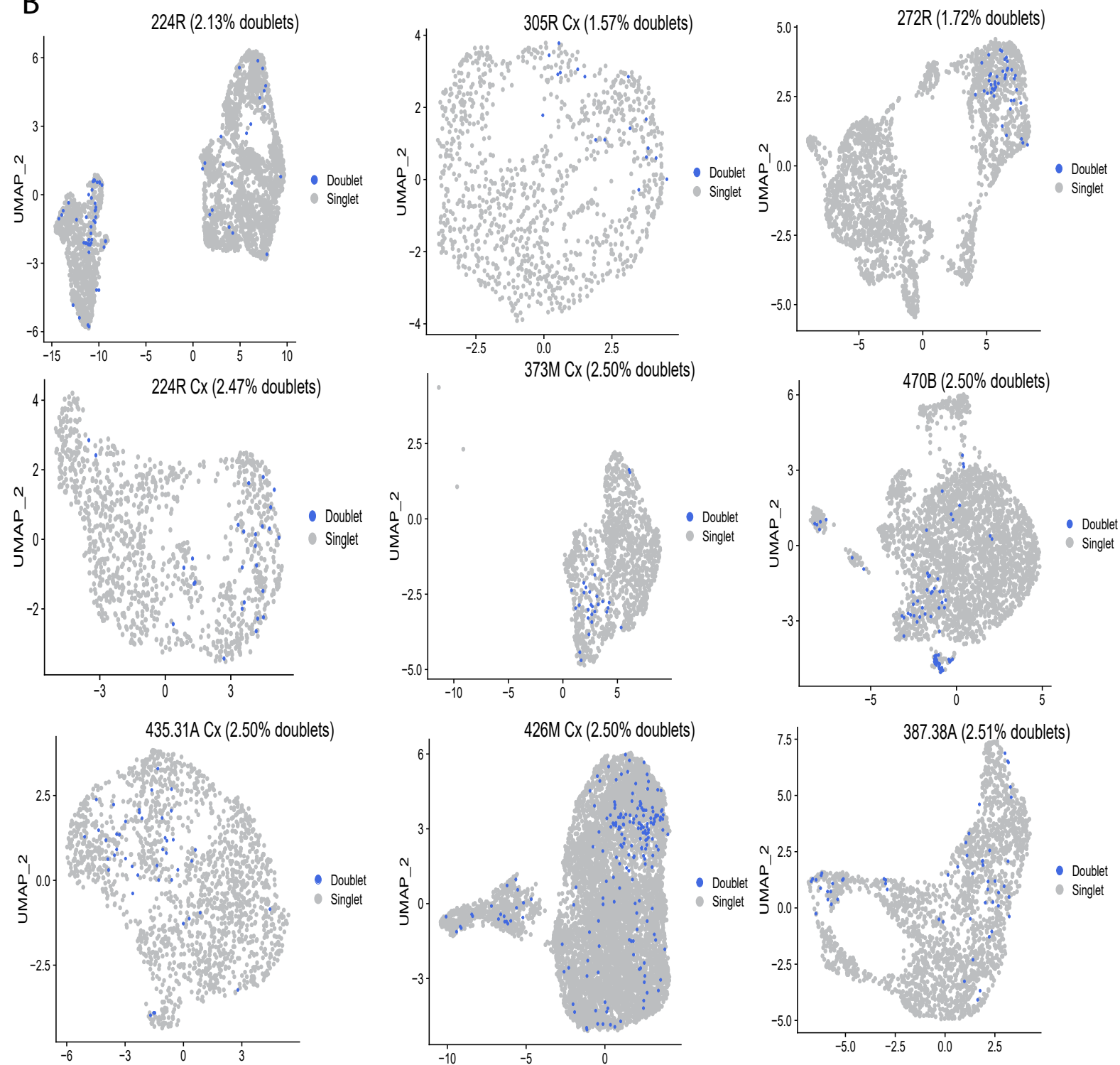

Supplement: Supplementary file 1 — Fig. S1. Doublet detection. (A) Percentage of doublets detected per sample. (B) Visualization of doublets in a UMAP per sample. [file MOL2-19-2776-s014.pdf]

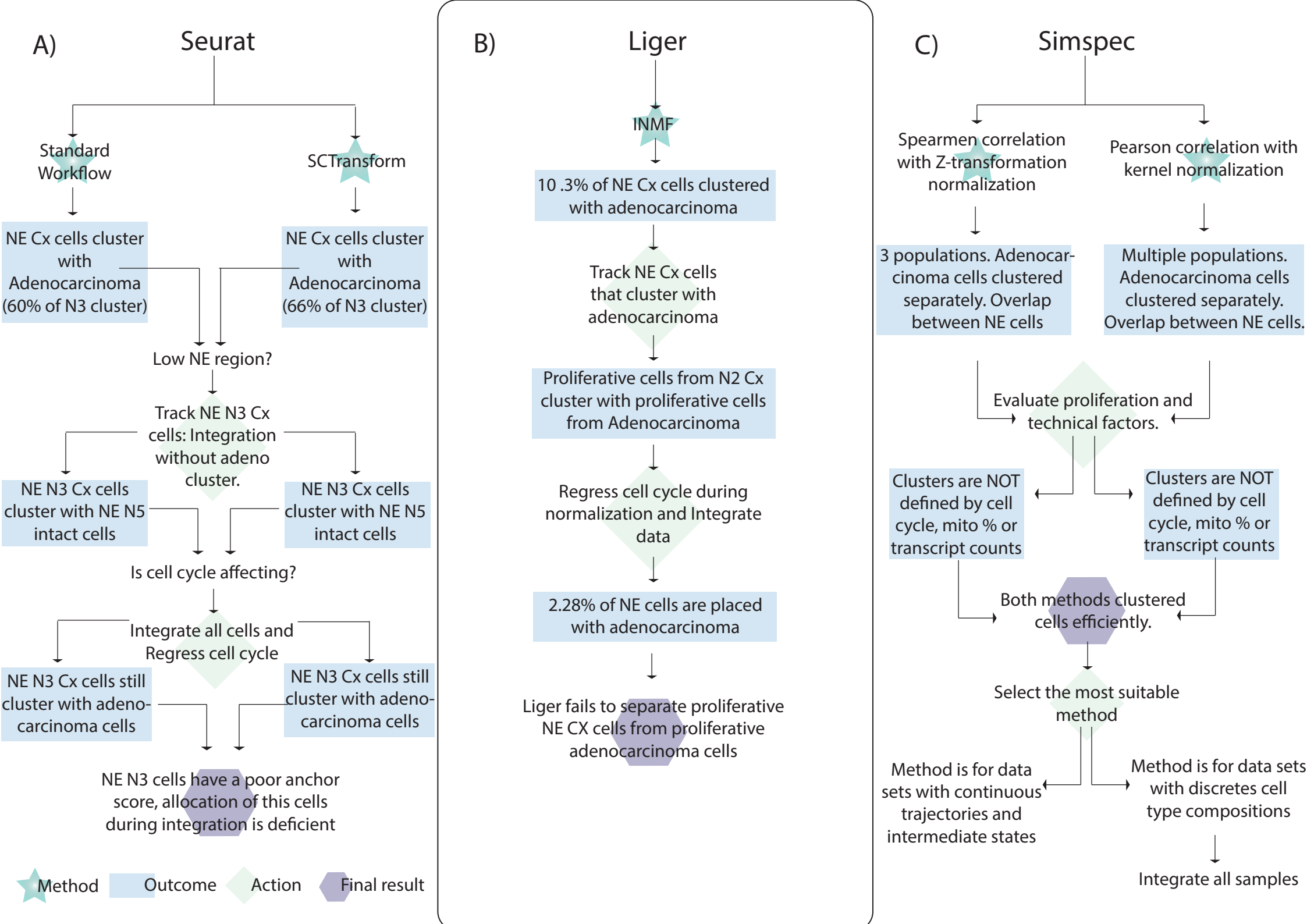

Supplement: Supplementary file 2 — Fig. S2. Workflow chart of benchmarking process. (A) Seurat integration workflow process. Seurat has two ways of pre‐processing the data for integration; standard workflow and SCTransfrom. Standard workflow includes normalization, finding variable features and scaling data. SCTransfrom is a wrapper that includes the standard workflow, but the data is not scaled. (B) Liger integration workflow process. Integrative non‐negative matrix factorization (iNMF) method was used. (C) Simspec integration workflow process. The cluster similarity spectrum method was used. Two correlation methods might be used: Pearson or spearman. Pearson correlation uses kernel normalisation and Spearman correlation uses Z‐transformation normalisation. [file MOL2-19-2776-s009.pdf]

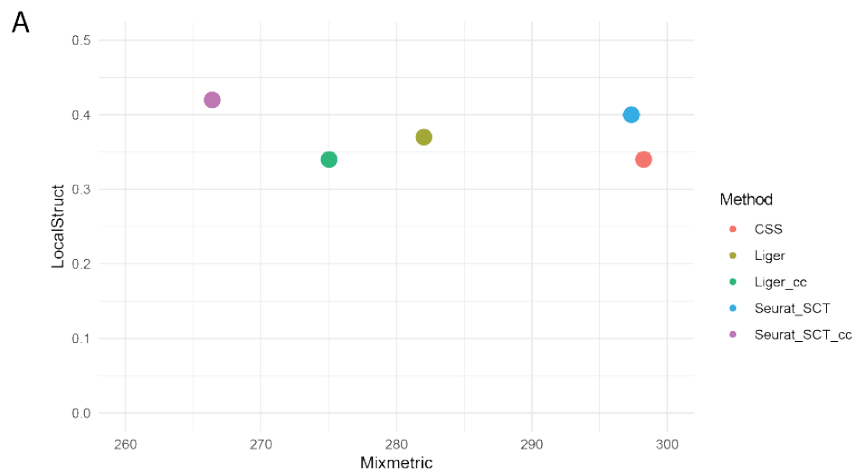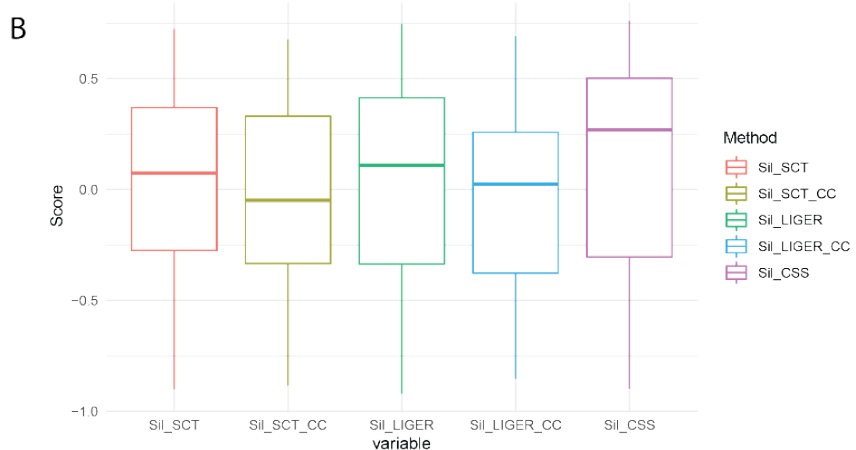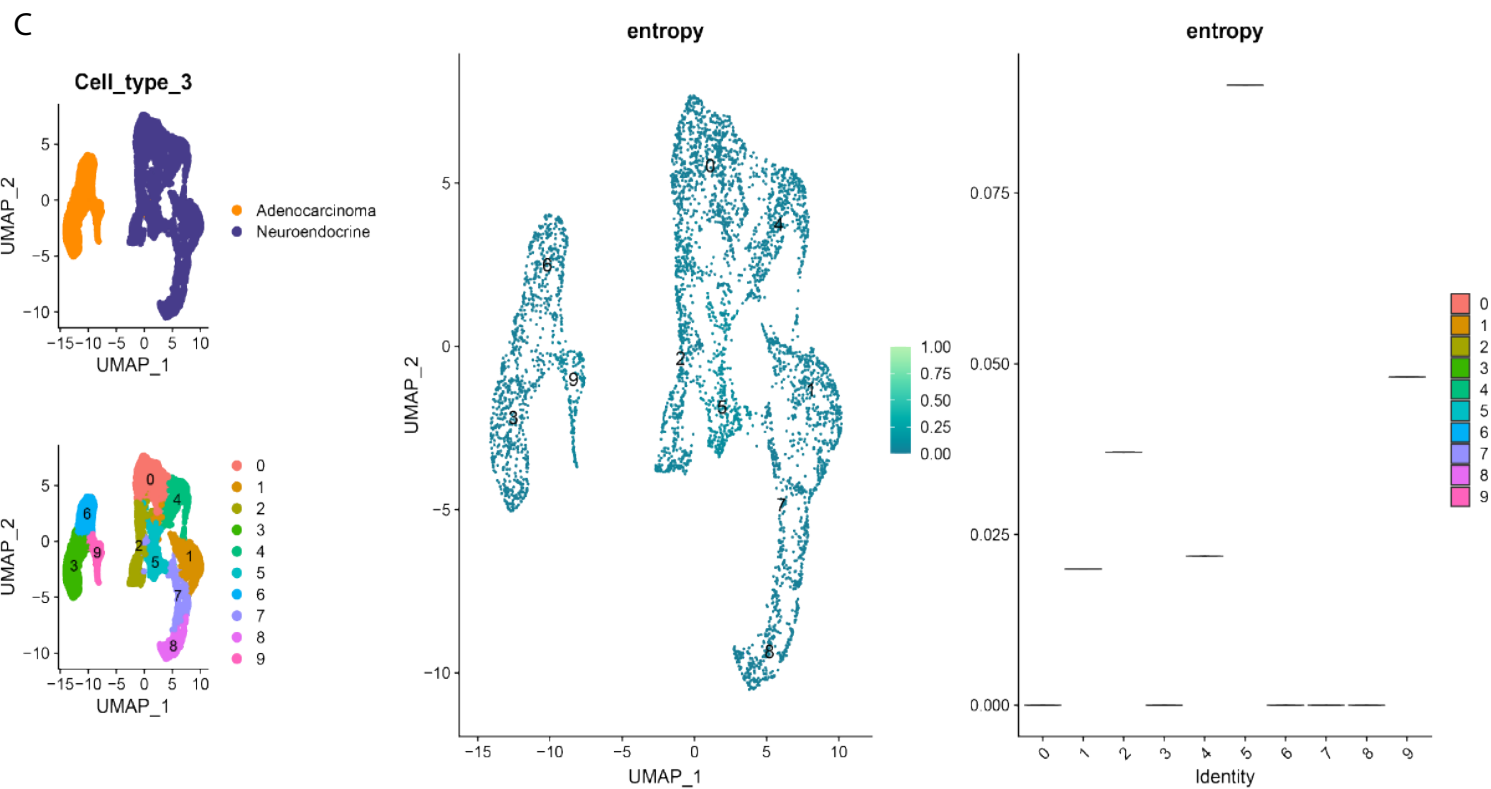

Supplement: Supplementary file 4 — Fig. S4. Metrics for evaluating integration performance. (A) Local structure and Mix metric depicted in a dotplot. (B) Boxplot of AWS scores per cell resulting from each integration method. (C) UMAPs depicting cell type (Adenocarcinoma and neuroendocrine), clusters, and entropy score. Violin plot showing the score per cluster. [file MOL2-19-2776-s003.pdf]

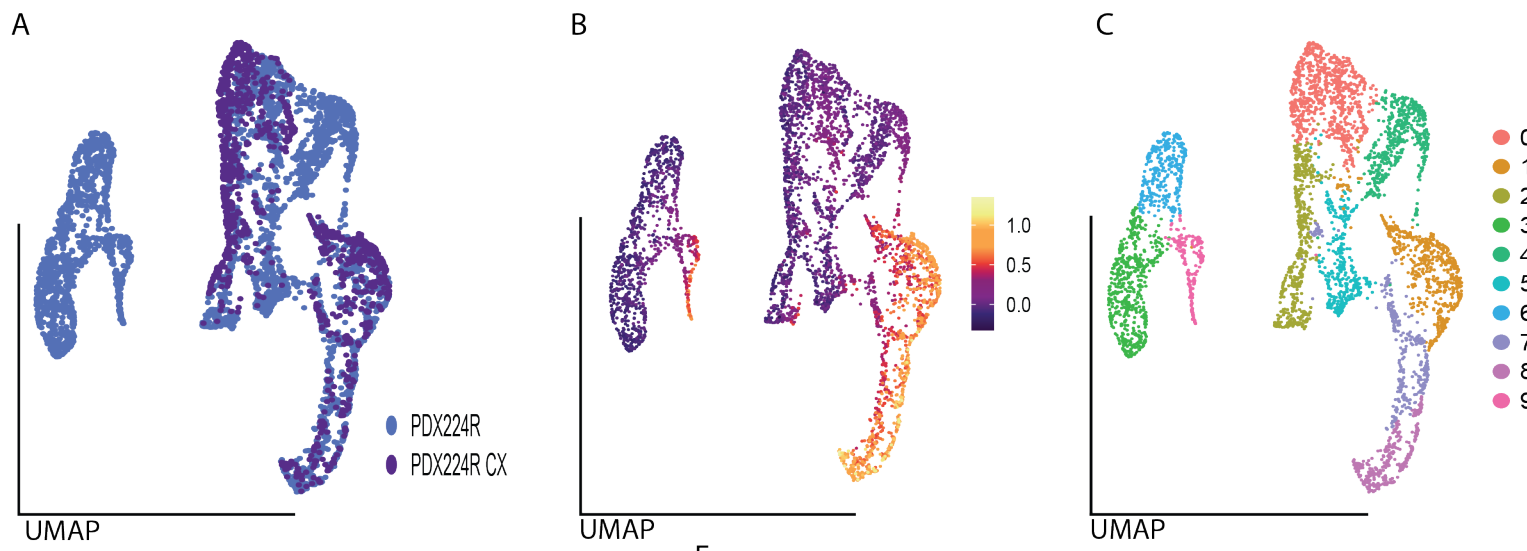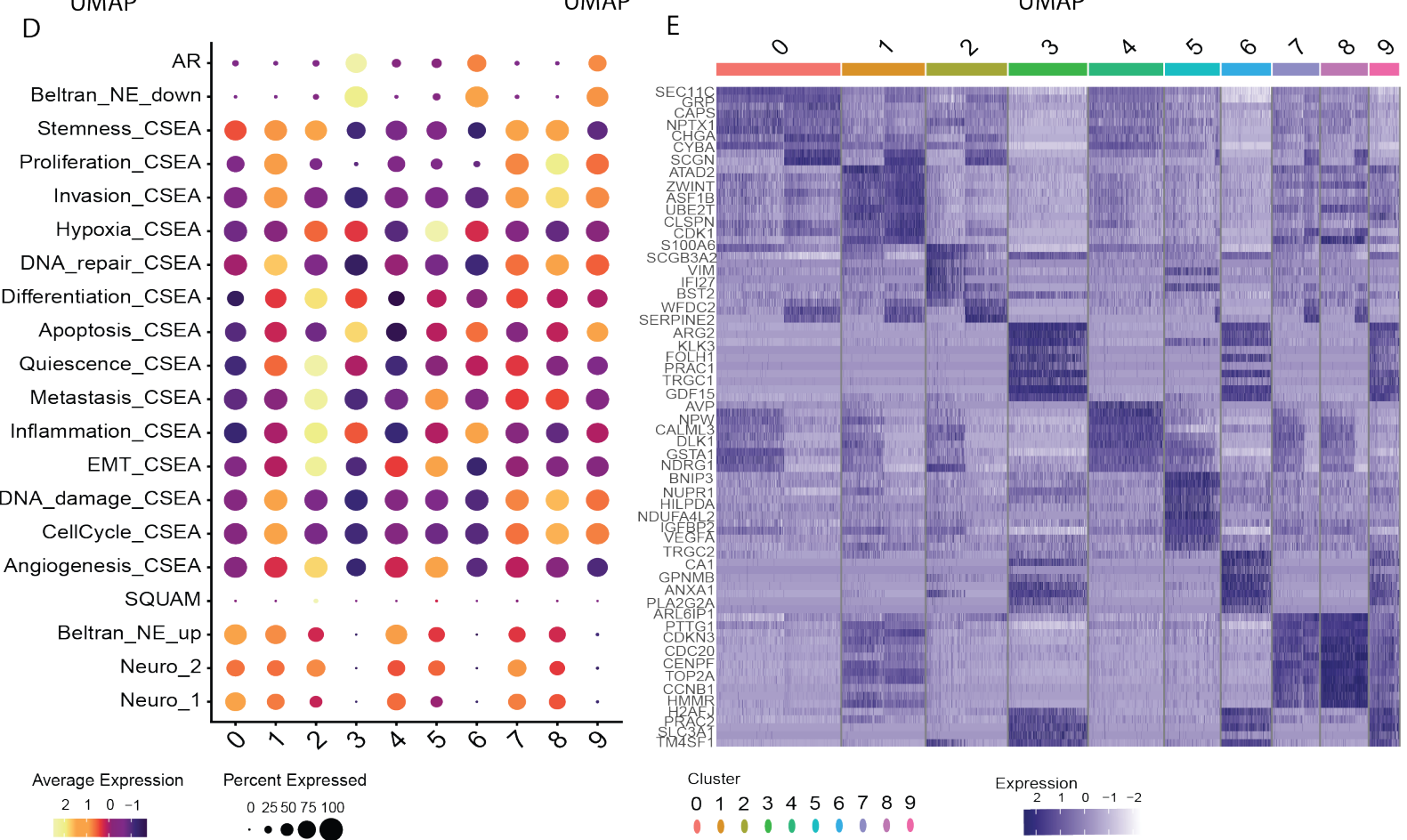

Supplement: Supplementary file 5 — Fig. S5. Tumour heterogeneity is preserved after integration with CSS. (A) UMAP representing the integration of PDX224R and PDX 224R‐Cx. (B) UMAP depicting proliferative cells. A score close to one indicates a high expression of proliferative genes. (C) UMAP showing the 10 clusters formed after integration. Clusters were defined with the same method used on previous chapter. (D) Cancer CEA, NEURO I‐II, Squamous and AR responsive signatures. The ratio of the circle represents the proportion of cells that express such signature, the colour of the circle represents the level of expression, yellow represents a high expression, purple represents a low expression. (E) Heatmap showing the top markers per cluster. [file MOL2-19-2776-s006.pdf]

A

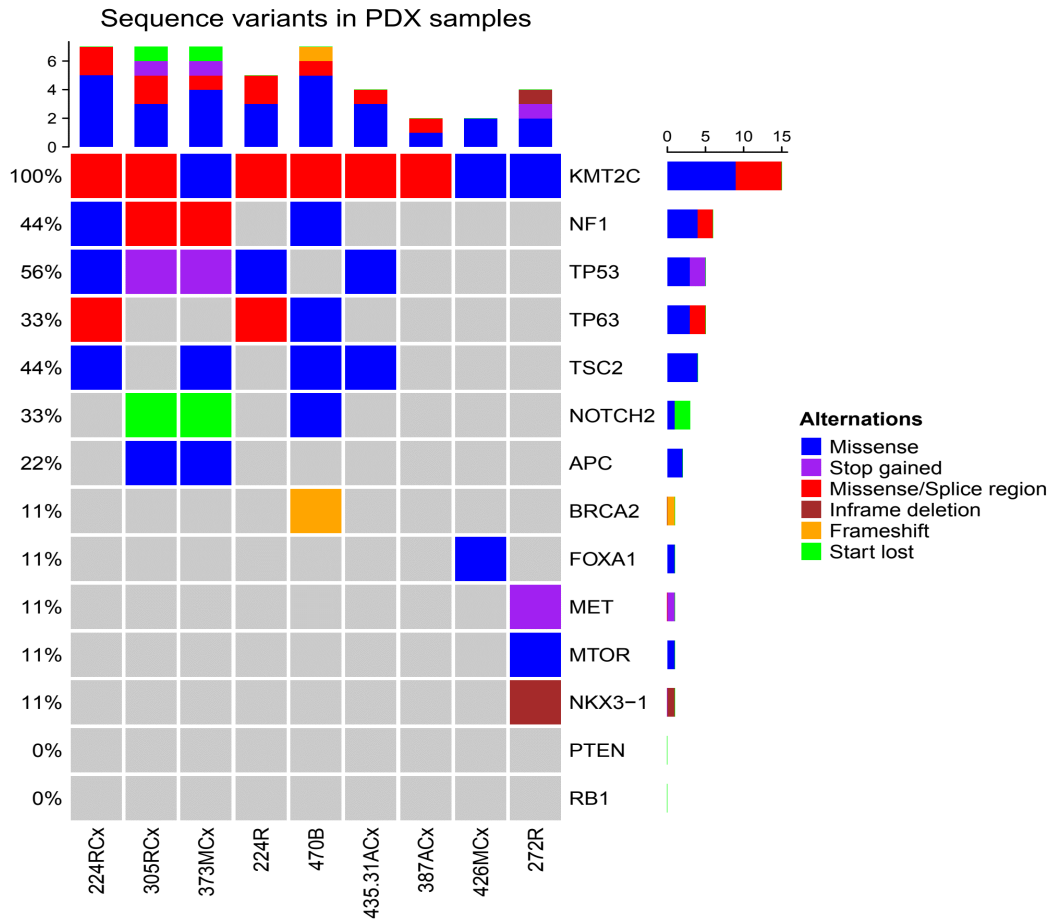

B

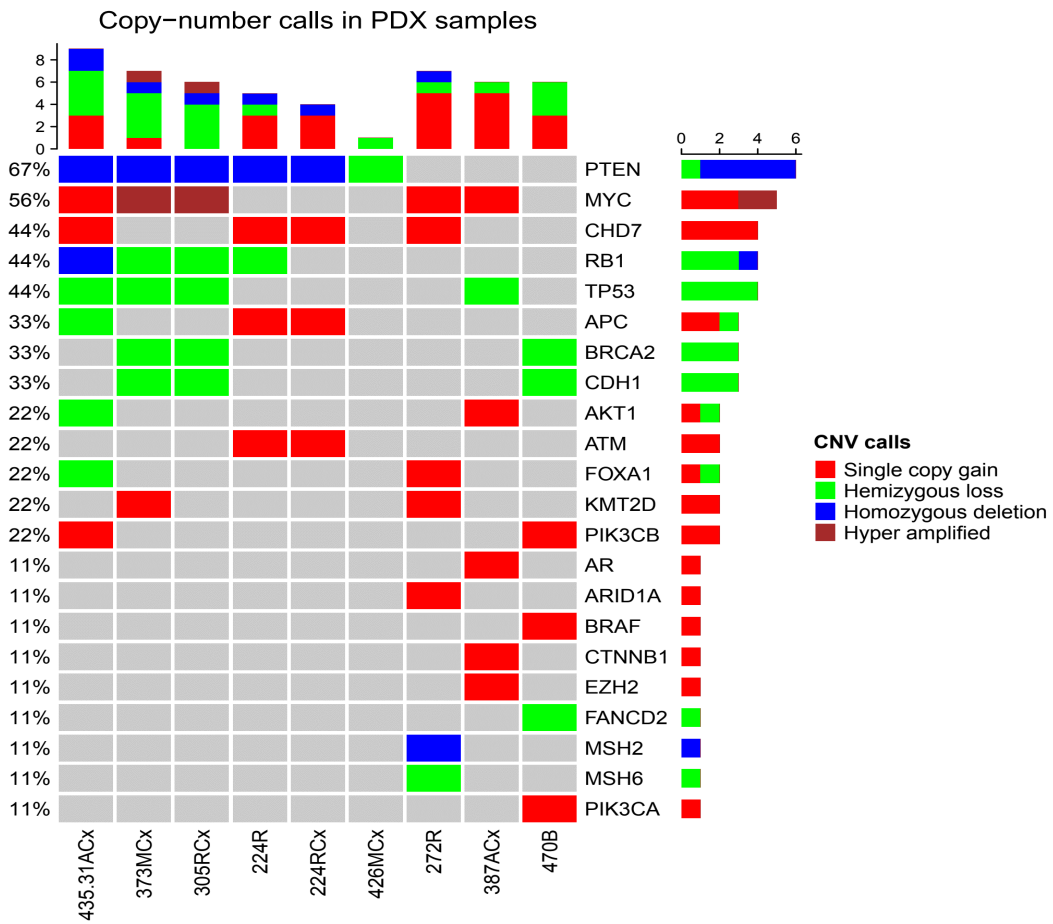

Supplement: Supplementary file 6 — Fig. S6. Mutational profiles of the patient‐derived xenograft models used in this study. (A) Protein‐coding mutations in selected cancer driver genes for each PDX model, coloured by effect on protein sequence. Sidebar displays overall frequency of mutations to each gene across the 9 PDXs. (B) Copy‐number aberrations in selected cancer driver genes for each PDX model coloured by magnitude of change relative to diploid. [file MOL2-19-2776-s007.pdf]

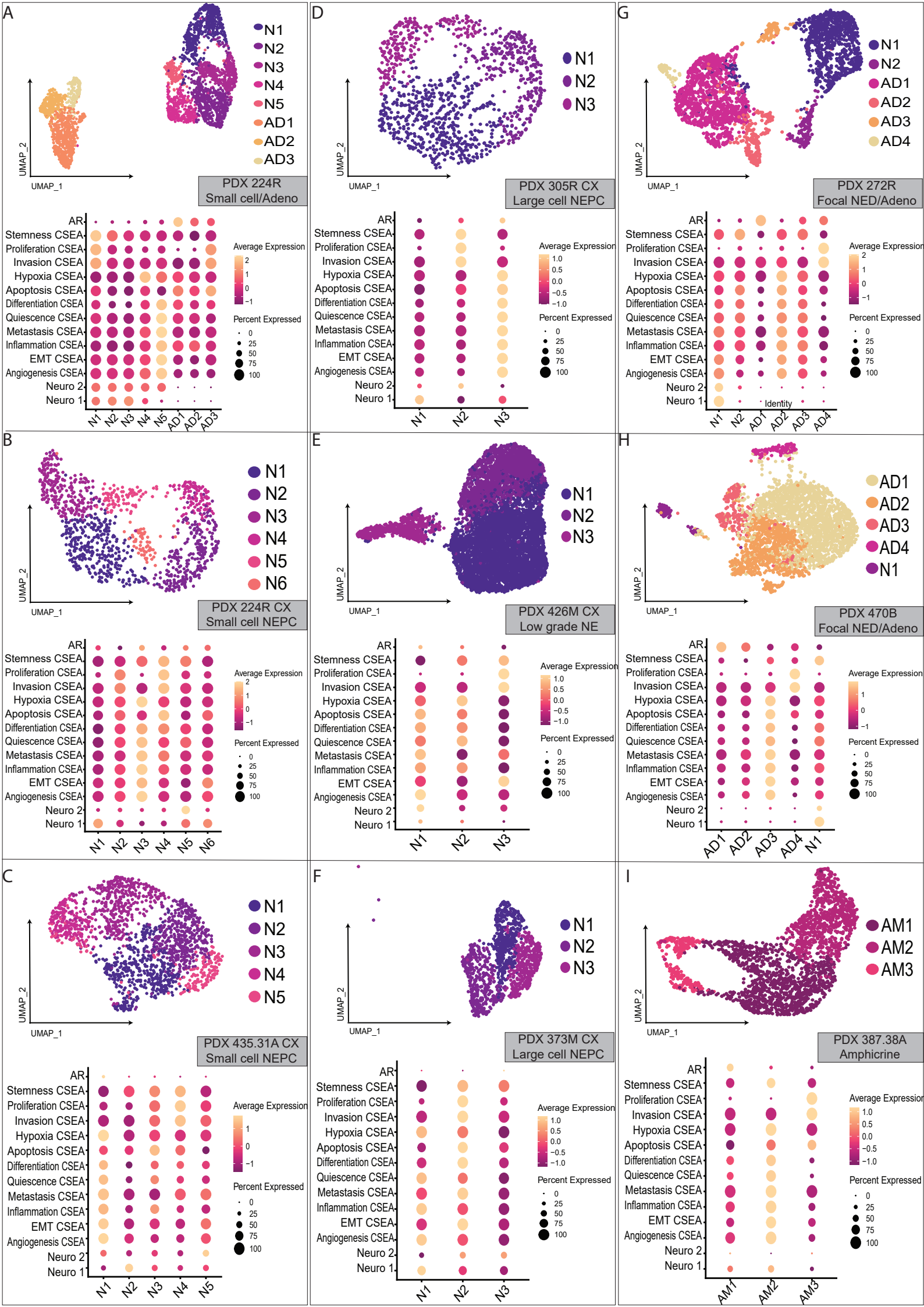

Supplement: Supplementary file 7 — Fig. S7. Functional state enrichment and transcriptional classification of single‐cell clusters across NEPC PDX models. UMAPs displaying cell clusters identified in each PDX, with corresponding gene set enrichment scores for functional states from the Cancer Single‐cell Expression Atlas (CSEA) (Yuan et al., 2018) and androgen receptor (AR) signaling and neuroendocrine prostate cancer (Neuro1 and Neuro2) signatures from Labrecque et al. (2019). Clusters are labelled according to whether cells express neuroendocrine markers (N), adenocarcinoma markers (AD) or both sets of markers (AM). PDXs are divided according to pathology. (A) mixed small cell‐adenocarcinoma PDX; (B) Small cell neuroendocrine prostate cancer (NEPC) PDX 224R‐Cx; (C) Small cell NEPC PDX 435.31A‐Cx; (D) Large cell NEPC PDX 305‐Cx; (E) Low‐grade neuroendocrine PDX 426M‐Cx; (F) Large cell NEPC PDX 373M‐Cx; (G) Adenocarcinoma with neuroendocrine differentiation (Adeno‐NED) PDX 272R; (H) Adeno‐NED PDX 470B; (I) Amphicrine PDX 387.38A. [file MOL2-19-2776-s001.pdf]

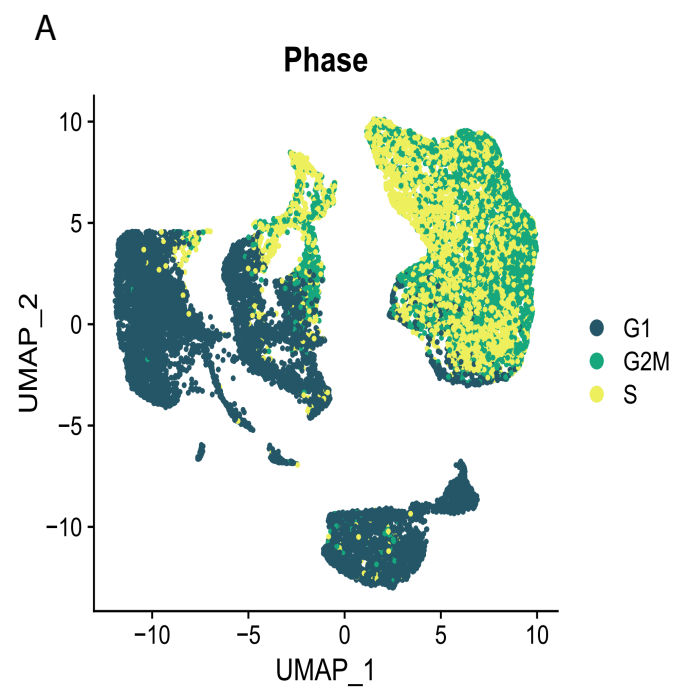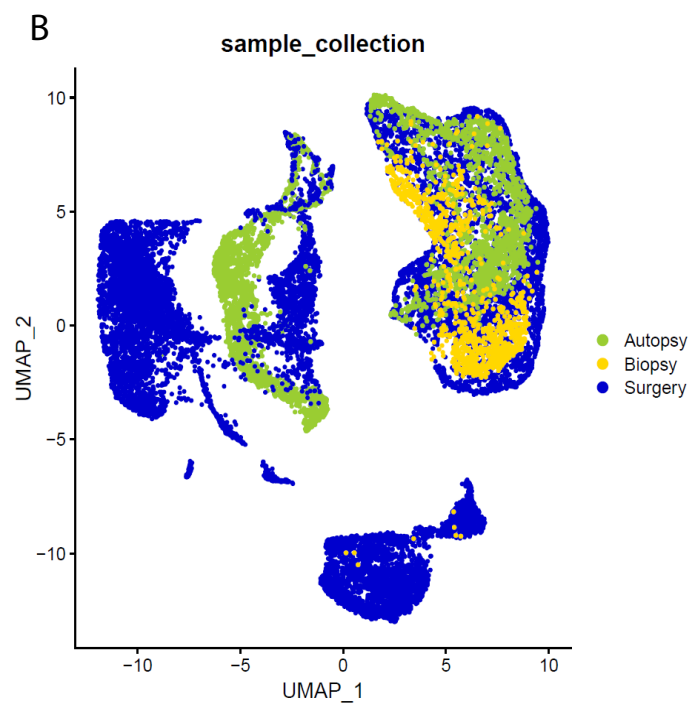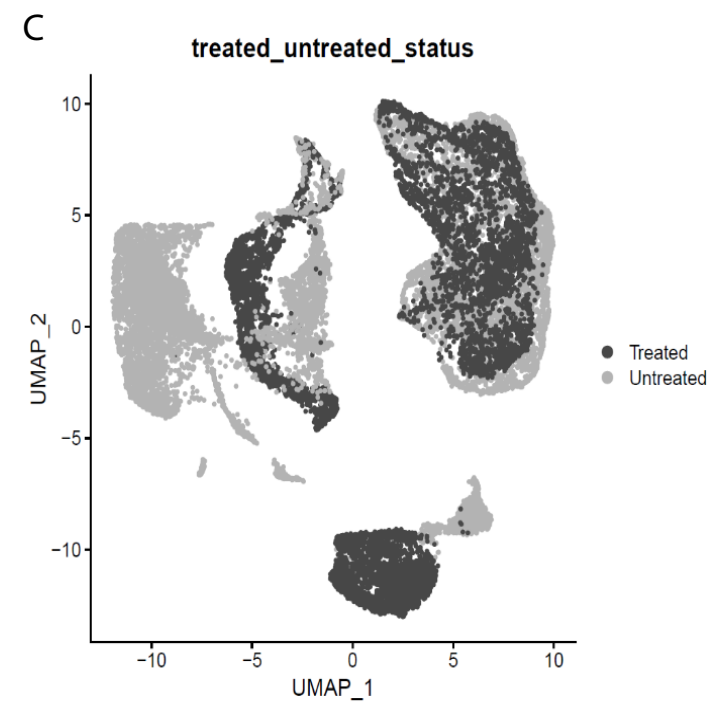

Supplement: Supplementary file 8 — Fig. S8. UMAPs of integrated data from all 9 PDXs with cells coloured by conditions. (A) Cell cycle phase, (B) site of collection or (C) treatment status. [file MOL2-19-2776-s011.pdf]

**A**

272R

470B

AR

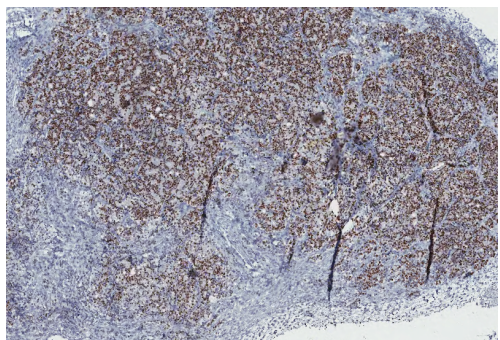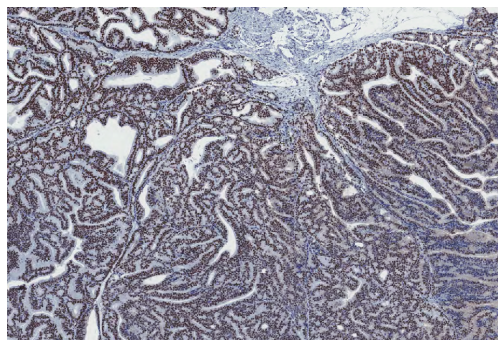

CD56

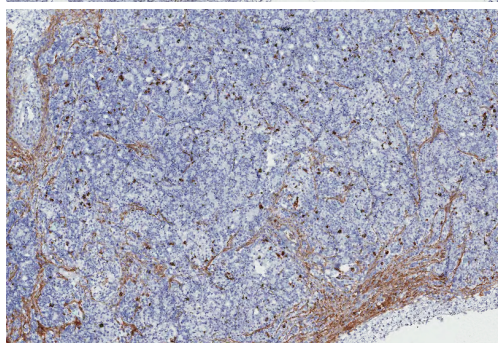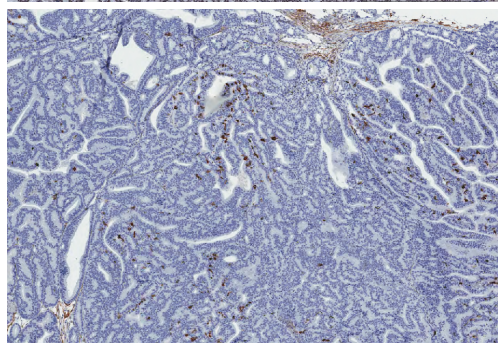

CHGA

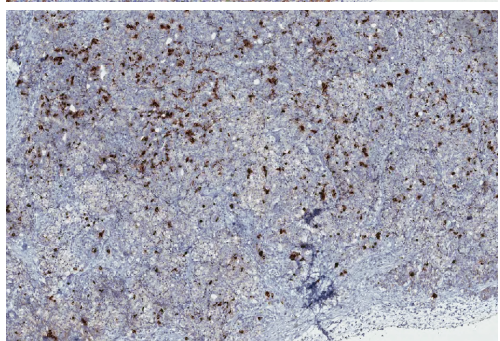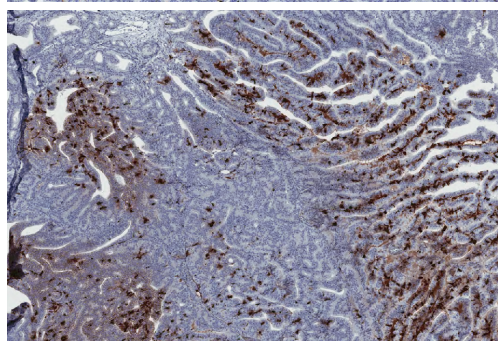

SYP

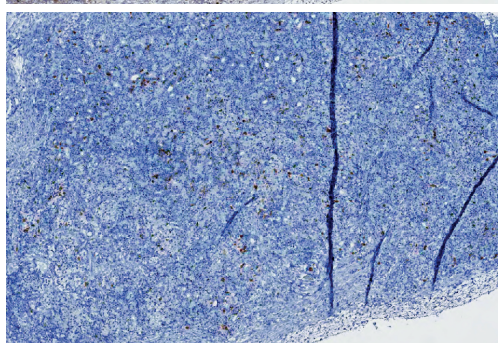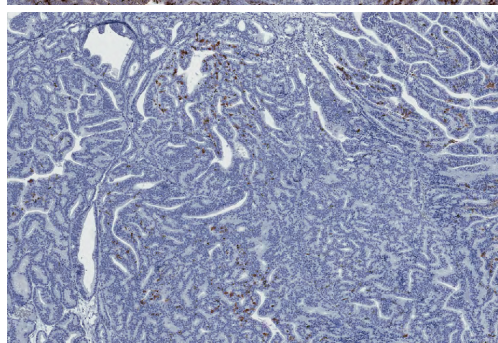

Scale 500 um

Supplement: Supplementary file 9 — Fig. S9. Whole tissue section to illustrate the ratio of NED vs adenocarcinoma. Scale bar = 500 μm. [file MOL2-19-2776-s010.pdf]

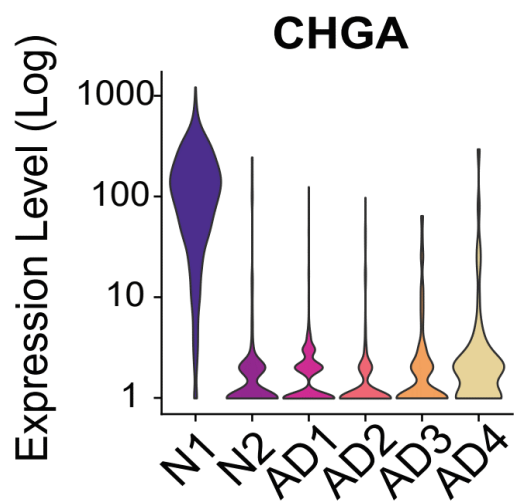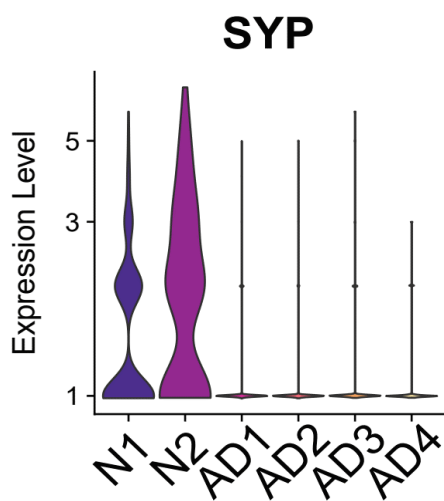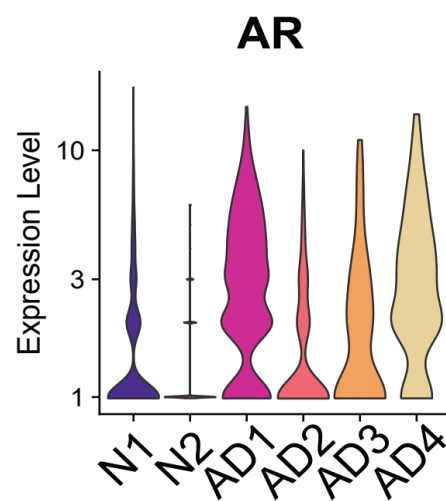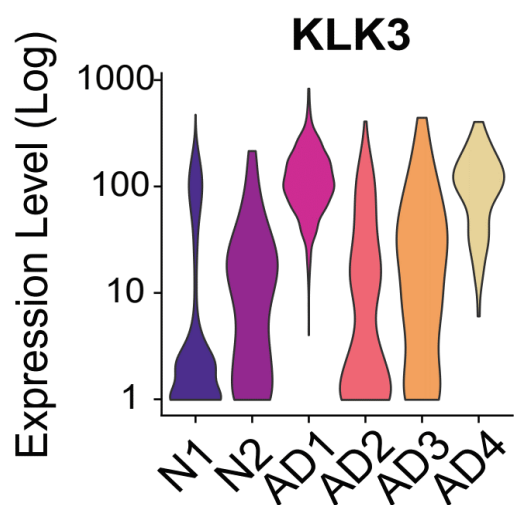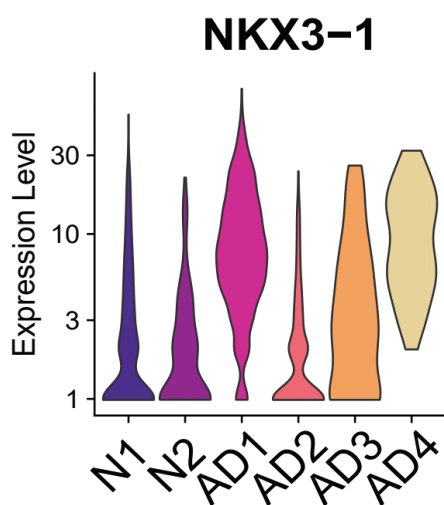

Supplement: Supplementary file 10 — Fig. S10. Expression of neuroendocrine markers genes in PDX 272R. Neuroendocrine and the adenocarcinoma marker genes expression by cluster in PDX 272R. [file MOL2-19-2776-s005.pdf]

**AR**

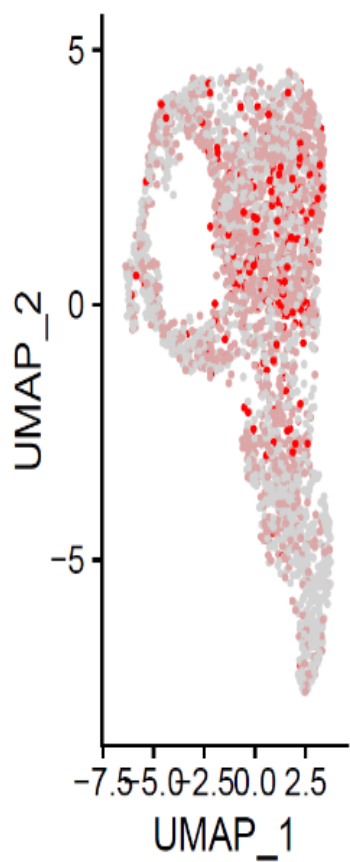

**SYP**

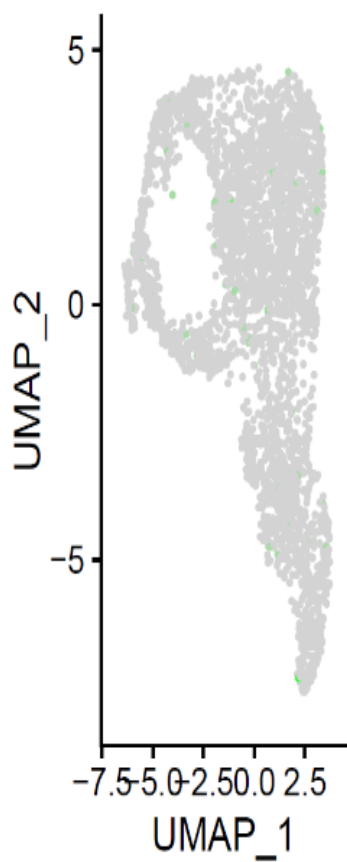

**AR\_SYP**

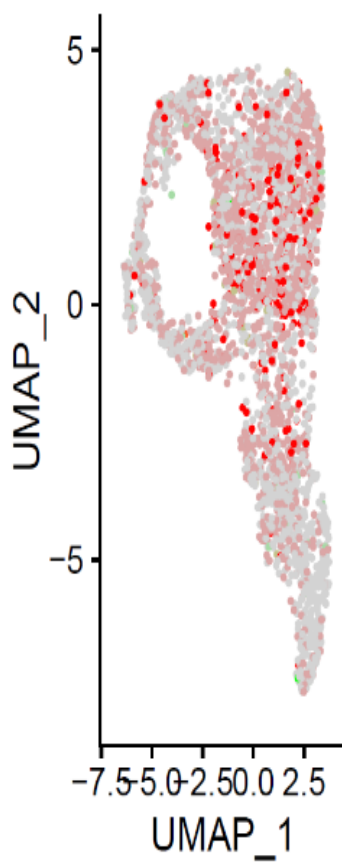

**Color threshold:**

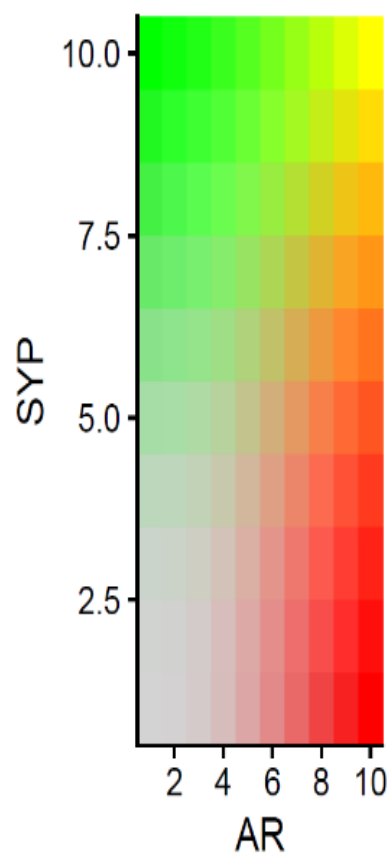

Supplement: Supplementary file 11 — Fig. S11. Co‐expression of the androgen receptor (AR) and synaptophysin (SYP) genes in tumour cells from PDX 287R. [file MOL2-19-2776-s008.pdf]

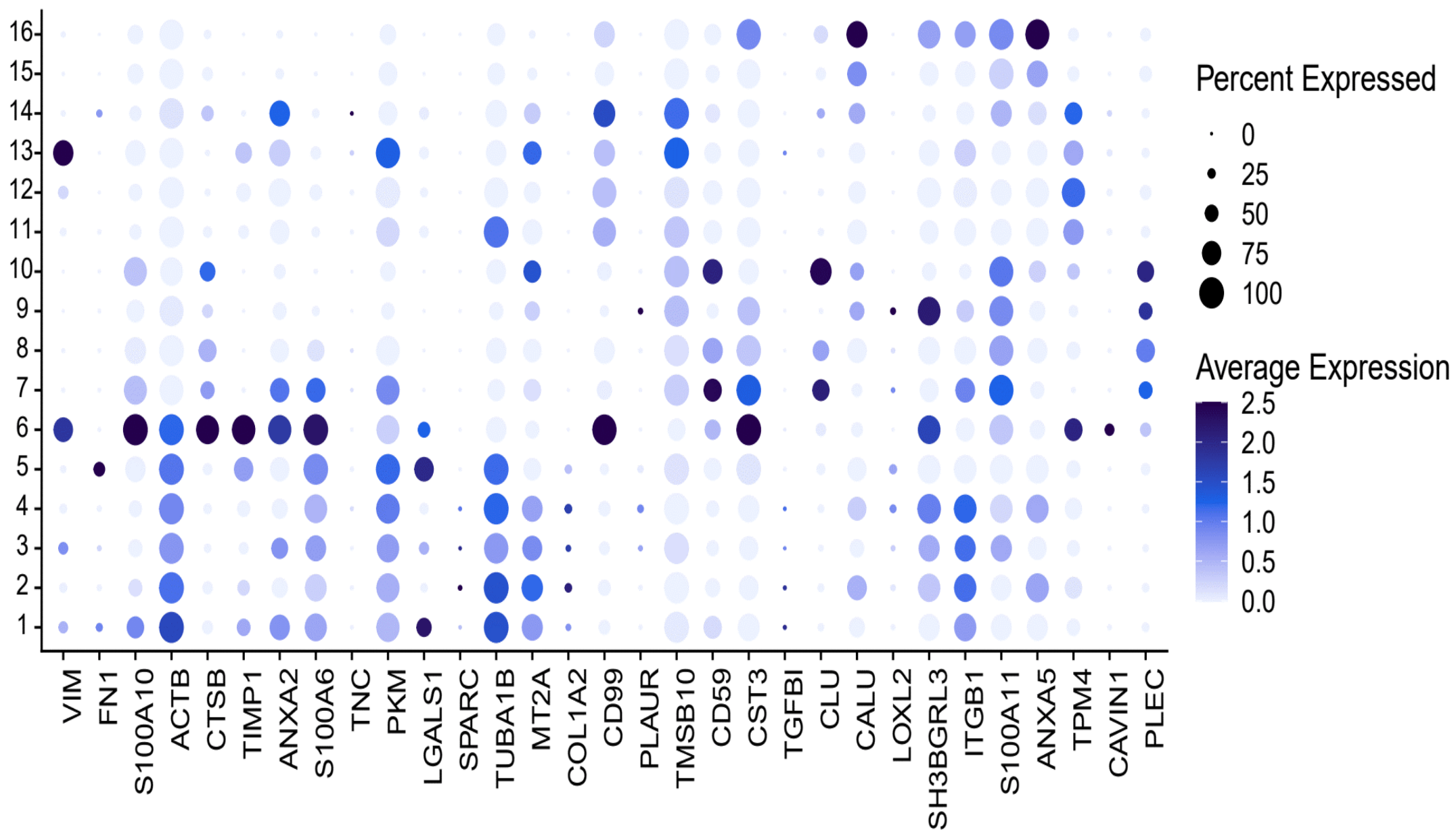

Supplement: Supplementary file 12 — Fig. S12. Expression of genes from the epithelial–mesenchymal transition gene set. Gene set from the cancer single‐cell expression atlas (Cancer SEA) across UMAP clusters from the integrated analysis of PDX data. [file MOL2-19-2776-s016.pdf]

# HOXB13

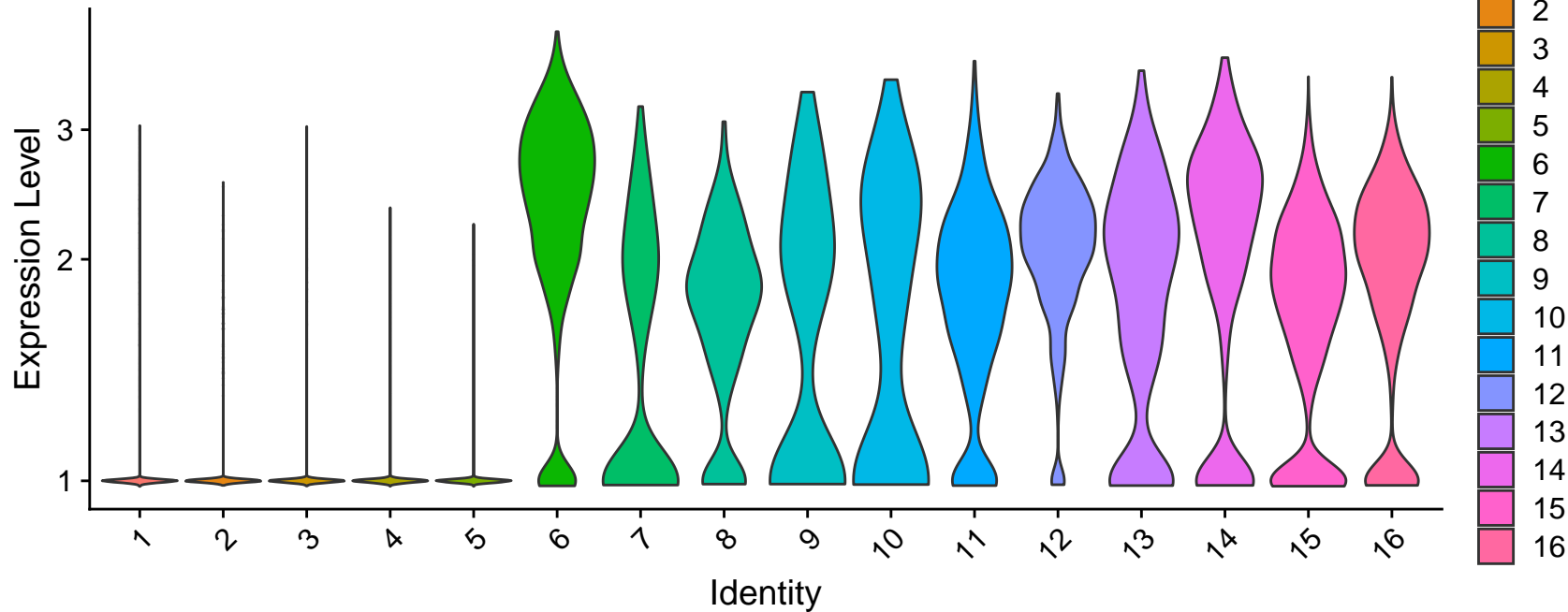

Supplement: Supplementary file 13 — Fig. S13. Expression of HOXB13 across clusters. [file MOL2-19-2776-s012.pdf]

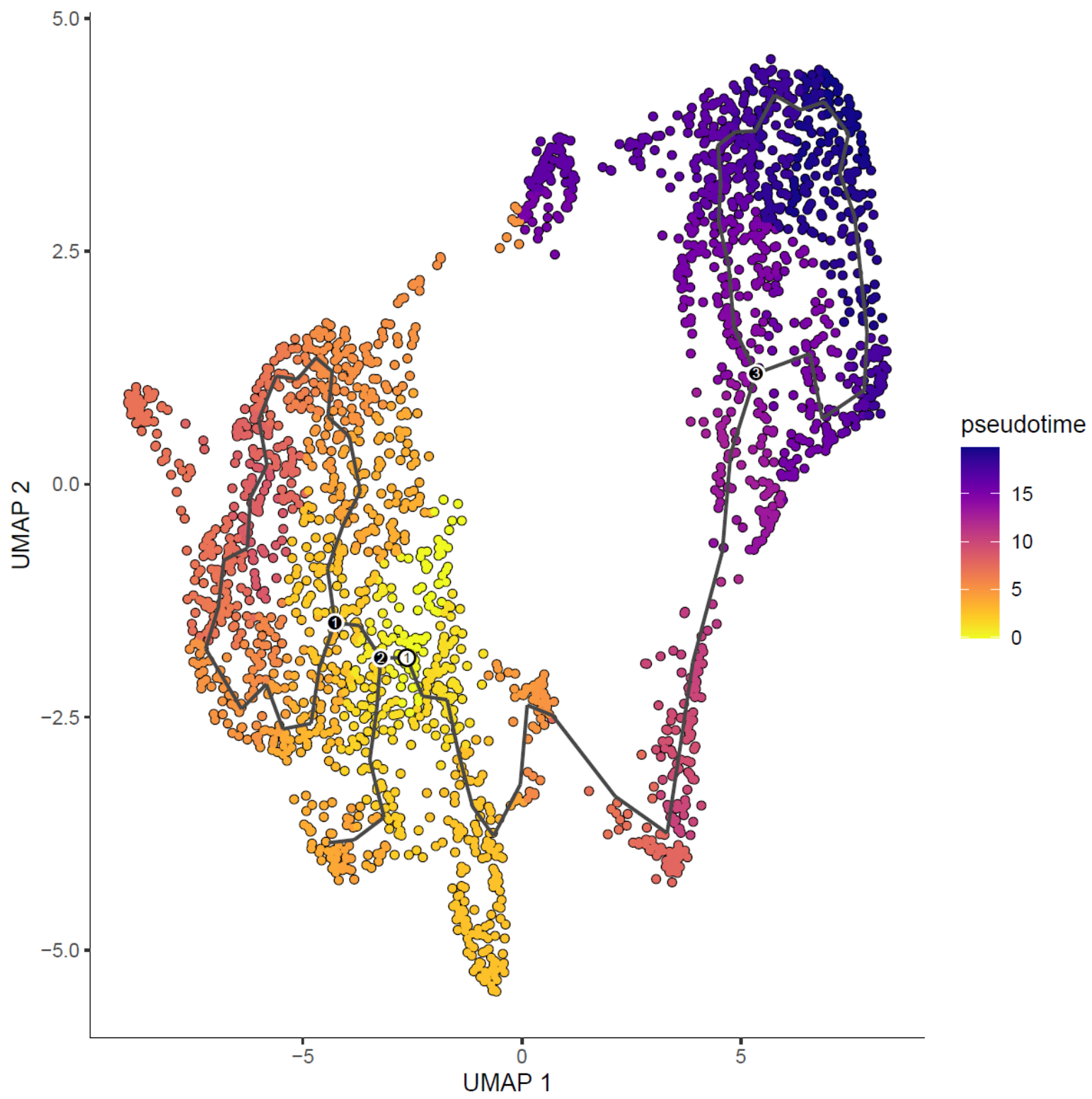

Supplement: Supplementary file 14 — Fig. S14. Pseudotime trajectory (Monocle 2) for tumour cells in PDX 272. Trajectory begins in the adenocarcinoma component (left; yellow/orange) and continues into the neuroendocrine component (right; blue/purple). [file MOL2-19-2776-s004.pdf]

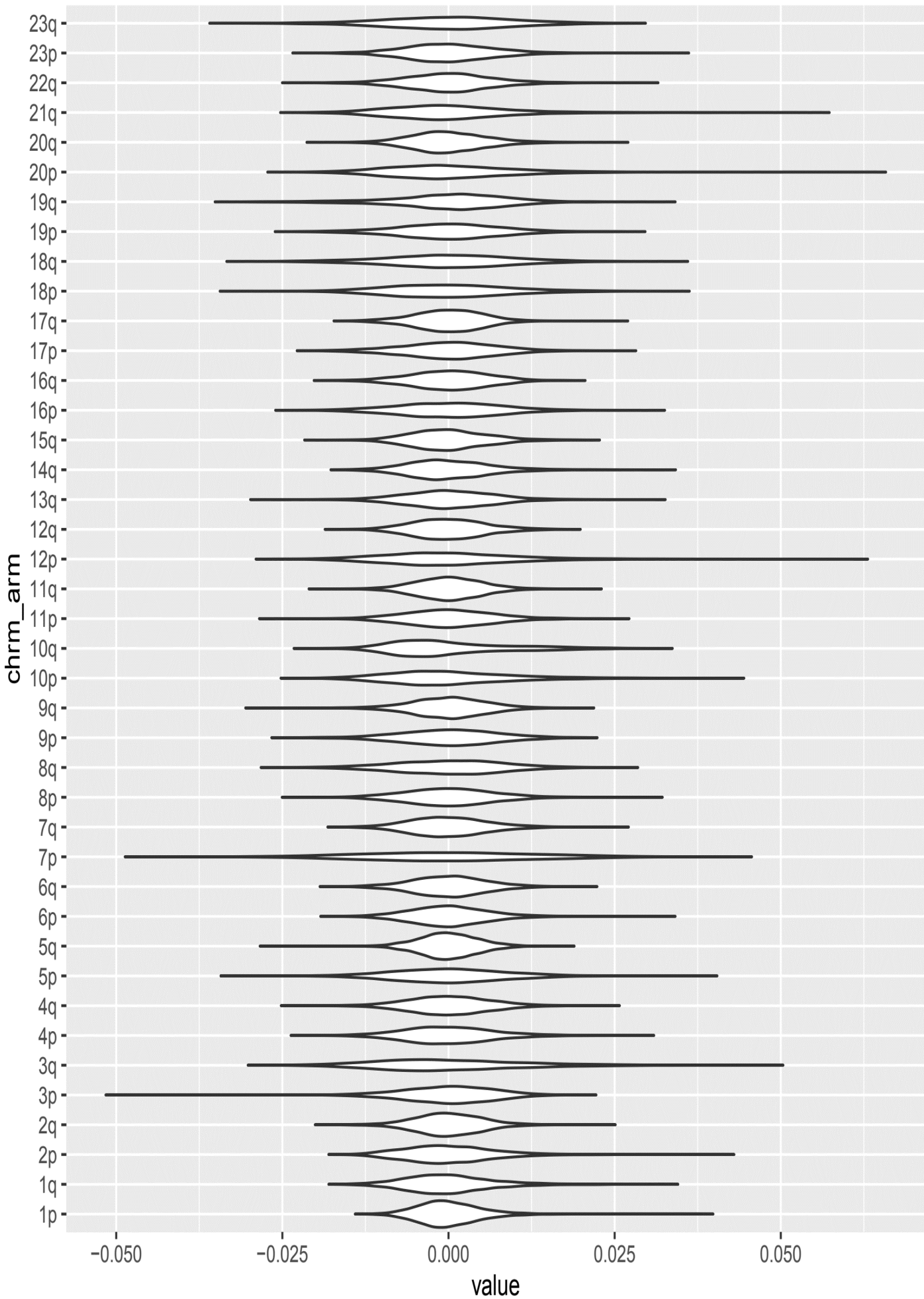

Supplement: Supplementary file 15 — Fig. S15. Relative expression of genes on each chromosome arm for tumour cells from PDX 470B. [file MOL2-19-2776-s015.pdf]
