# Supplementary material for: Single‐cell transcriptomics redefines focal neuroendocrine differentiation as a distinct prostate cancer pathology
Source: Mol Oncol. 2025 Jul 24;19(10):2776–96. doi: 10.1002/1878-0261.70099 (PMC12515717; doi:10.1002/1878-0261.70099)
Supplement: Supplementary file 3 — Fig. S3. Integration outcomes of 3 different pipelines. UMAPS represents the outcome of each step of the process (A) Seurat pipeline. (A1) UMAP representing the Seurat integration using SCTransform as a normalisation method. In a green outline, castrated cells cluster with adenocarcinoma. (A2) UMAP shows the main subpopulation of castrated cells that cluster with adenocarcinoma. (A3) UMAP represents the integration after removing the cell cycle phase as a source of variation. Castrated cells from cluster N3 remain clustering with adenocarcinoma and are outlined in green. (A4) UMAP represents the outcome of integration with Seurat. (B) Liger pipeline. (B1) UMAP representing the Liger integration. In a green outline, castrated cells cluster with adenocarcinoma. (B2) UMAP shows the main subpopulation of castrated cells that cluster with adenocarcinoma—outlined in green castrated N2 cells. (B3) UMAP represents the integration after removing the cell cycle as a source of variation. Castrated N1 and N3 cells clustered with adenocarcinoma, outlined in green. (B4) UMAP showing the outcome of integration with Liger. C) Simspec CSS pipeline. (C1) UMAP represents the initial outcome of the integration using Simspec. Circled in a green outline, a cluster is uniquely formed by castrated cells. (C2) UMAP coloured by the expression of mitochondrial percentage. (C3) UMAP coloured by the transcript (UMI) count. (C4) UMAP with the Simspec integration pipeline after QC. [file MOL2-19-2776-s013.pdf]

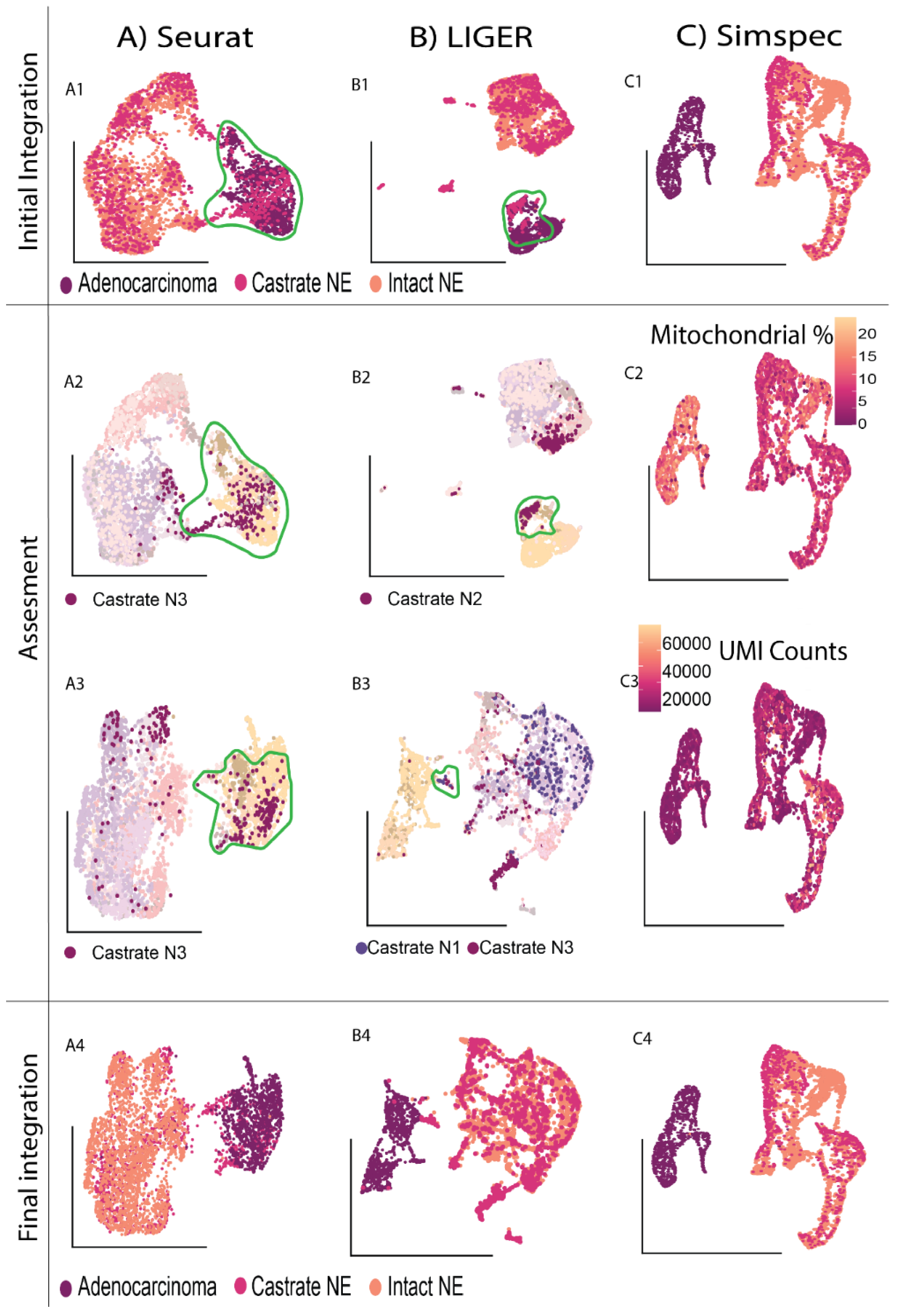
A) Seurat
B) LIGER
C) Simspec
Initial Integration
A1
B1
C1
Adenocarcinoma
Castrate NE
Intact NE
Assesment
Mitochondrial %
20
15
10
5
0
A2
B2
C2
Castrate N3
Castrate N2
60000
40000
20000
UMI Counts
A3
B3
C3
Castrate N3
Castrate N1
Castrate N3
Final integration
A4
B4
C4
Adenocarcinoma
Castrate NE
Intact NE
